# Fungal genomes: suffering with functional annotation errors

**DOI:** 10.1186/s43008-021-00083-x

**Published:** 2021-11-01

**Authors:** Tapan Kumar Mohanta, Ahmed Al-Harrasi

**Affiliations:** Natural and Medical Sciences Research Center, University of Nizwa, 616 Nizwa, Oman

**Keywords:** Fungal genome, Fungi, Genome, Annotation, Selenoprotein, WRKY, Calcium signaling, Calcium dependent protein kinase

## Abstract

**Background:**

The genome sequence data of more than 65985 species are publicly available as of October 2021 within the National Center for Biotechnology Information (NCBI) database alone and additional genome sequences are available in other databases and also continue to accumulate at a rapid pace. However, an error-free functional annotation of these genome is essential for the research communities to fully utilize these data in an optimum and efficient manner.

**Results:**

An analysis of proteome sequence data of 689 fungal species (7.15 million protein sequences) was conducted to identify the presence of functional annotation errors. Proteins associated with calcium signaling events, including calcium dependent protein kinases (CDPKs), calmodulins (CaM), calmodulin-like (CML) proteins, WRKY transcription factors, selenoproteins, and proteins associated with the terpene biosynthesis pathway, were targeted in the analysis. Gene associated with CDPKs and selenoproteins are known to be absent in fungal genomes. Our analysis, however, revealed the presence of proteins that were functionally annotated as CDPK proteins. However, InterproScan analysis indicated that none of the protein sequences annotated as “calcium dependent protein kinase” were found to encode calcium binding EF-hands at the regulatory domain. Similarly, none of a protein sequences annotated as a “selenocysteine” were found to contain a Sec (U) amino acid. Proteins annotated as CaM and CMLs also had significant discrepancies. CaM proteins should contain four calcium binding EF-hands, however, a range of 2–4 calcium binding EF-hands were present in the fungal proteins that were annotated as CaM proteins. Similarly, CMLs should possess four calcium binding EF-hands, but some of the CML annotated fungal proteins possessed either three or four calcium binding EF-hands. WRKY transcription factors are characterized by the presence of a WRKY domain and are confined to the plant kingdom. Several fungal proteins, however, were annotated as WRKY transcription factors, even though they did not contain a WRKY domain.

**Conclusion:**

The presence of functional annotation errors in fungal genome and proteome databases is of considerable concern and needs to be addressed in a timely manner.

**Supplementary Information:**

The online version contains supplementary material available at 10.1186/s43008-021-00083-x.

## Background

The term “gene annotation” refers to detailed information provided on a specific gene and its translated protein products (Curwen et al. 2004). Gene annotation of an uncharacterized gene/protein is typically conducted using homology-based sequence similarity, or reference-based annotation methods (Cai and Bork 1998; Taher et al. 2003; Meyer and Durbin 2004). These methods are the most widely-accepted approaches for gene annotation, and prediction. In some cases, a minor modification in homology-based annotation, however, can result in functional annotation errors, particularly in the case of fungi. Computationally-predicted annotation of genes can be subject to both false positive and negative errors.

A fundamental goal of current genome sequencing efforts is to provide universal availability of the genome, gene, and protein sequences of hundreds and thousands of organisms. This goal will enable the scientific community to investigate and correlate the genomic and molecular basis of gene/protein function and regulation, as well as their evolution. These data can be used to address many biological questions. There are several databases present in the public domain, including the National Center for Biotechnology Information (NCBI), UniProt/SwissProt (Consortium 2018), Ensembl (Hunt et al. 2018), and others, that provide genome, gene, and protein sequences of thousands of species, as well as other biological data on the regulation, expression, evolution, and phylogeny of genes and proteins. In addition, these databases also provide analytical tools to determine the homology of new gene/protein sequences. The sequence data entered into these databases are often annotated based on computationally-predicted protein functions. While information regarding the functional role of many genes/proteins are available, there is no guarantee regarding the accuracy of the predicted annotation. In fact, the rapid accumulation of sequence data at the current scale and breadth is rarely based on experimental validation. Computationally annotated sequences can be misannotated, which may in turn result in flawed experimentation, or at times, wasted efforts of functional validation. Therefore, the day-to-day propagation of errors needs to be addressed. The identification of genes and genome annotation for bacterial species is comparatively easy relative to the same processes in eukaryotes, since > 90% of the bacterial genome encodes protein coding sequences. Gene finding software analyzes genome sequences in six possible reading frames (3 forward and 3 reverse), which allows for highly accurate gene identification and rendering of protein sequence data. In eukaryotic systems (including plants, animals, and fungi), however, gene identification and annotation are difficult owing to the presence of non-coding intron sequences and the fact that genes are located far apart from each other on a linkage group. In addition, the presence of large eukaryotic genomes can make it challenging to assemble and annotate a genome. Therefore, the computationally-based automated method of gene and genome assembly and annotation is less accurate than it is in prokaryotic species. New genome sequence data are constantly being added, resulting in the rapid accumulation of data, which has been occurring at an astounding pace. Although the use of these data for experimental validation and the discovery of novel, beneficial products is very promising, their efficient and optimal utilization can be hampered if the sequence data are prone to false positive or negative annotations.

It is well known that fungi do not encode a “calcium dependent protein kinase” (CDPK) gene family in its genome. Fungal genomes, however, do contain calmodulins (CaM), and calmodulin like (CML) proteins. Fungi also do not encode selenoprotein containing the amino acid, selenocysteine (Mariotti and Guigó 2015) or WRKY transcription factors. Therefore, an analysis of 689 fungal species (7.15 million protein sequences) was conducted which targeted the presence of CDPK, selenocysteine/selenoprotein, CaM, CML, and WRKY transcription factors in fungi and their mis-annotation.

## Results

To determine the potential presence of CDPK genes/proteins in fungi, the annotated proteome files of 689 fungal species were downloaded from the NCBI website. The data file comprised approximately 7.15 million protein sequences of species across the fungal kingdom, including Ascomycota, Basidiomycota, Blastocladiomycota, Chytridiomycota, Glomeromycota, Microsporidia, Mucoromycota, Neocallimastigomycota, Opisthokonta, and Zoopagomycota. The species encode a range of 17–32,854 proteins. Most of the studied fungal species belonged to the phylum, Ascomycota (67.63%), followed by Basidiomycota (21.62%), and Microsporidia (3.77%). The presence of CDPK proteins were queried in the proteome files of these 689 species. The search resulted in the identification of 521 protein sequences in 197 species annotated as a “calcium/calmodulin-dependent protein kinase” (Additional file 1). Subsequently, 521 protein sequences annotated as a “calcium/calmodulin-dependent protein kinase” were analyzed using ScanProsite (Sigrist et al. 2012) and MEME suite (Bailey et al. 2009) software to identify the presence of kinase and EF-hand domains. The analysis revealed the presence of an N-terminal domain and a kinase domain in all of the protein sequences, however, none of the protein sequences contained an auto-inhibitory domain and a regulatory domain with four calcium binding EF-hands.

An analysis of calcium binding, calmodulin (CaM) and calmodulin-like (CML) proteins was also conducted. CaM and CML proteins are generally characterized by the presence of four calcium binding EF-hands (Mohanta et al. 2017) (Additional file 2). Analysis of the fungal protein dataset annotated as a “calmodulin protein” revealed the presence of 2–4 EF-hands, instead of a consistent four EF-hands (Additional file 3). A protein annotated as “calmodulin-binding protein Sha1” (accession XP_001269668.1, *Aspergillus clavatus* NRRL 1) was found to contain a cecropin family signature motif rather than a calmodulin binding EF-hand domain (Additional file 3). Similarly, a protein annotated as “IQ calmodulin-binding motif domain protein” (accession PKX94477.1, *Aspergillus novofumigatus*) did not possess any signature calmodulin motif/domain (Additional file 3). In a further search for calmodulin-like (CML) proteins in the fungal dataset, 14 CML proteins were identified among the 7.15 million protein sequences that were analyzed (Additional file 4). Prosite analysis of the CML proteins identified in fungi revealed the presence of two, three, and four EF-hands (Additional file 4). Based on the general consensus for CML proteins, however, they contain four calcium binding EF-hands, which was not the case in all of the fungal proteins annotated as CML proteins.

**Fig. 1:**
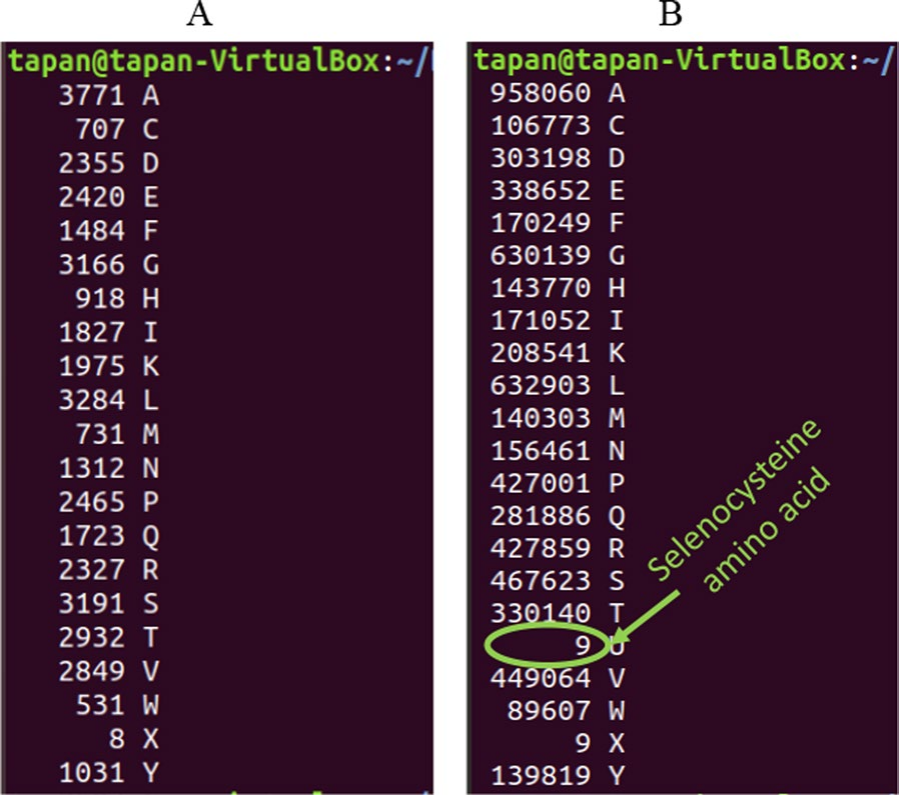
**A** Amino acid composition of fungal protein sequences annotated with the term selenoprotein/selenocysteine. No selenocysteine amino acids were detected in any of the protein sequences annotated as selenocysteine/selenoprotein. **B** Amino acid composition of the *Chlamydomonas reinhardtii* proteome revealing the presence of Sec (U) amino acids. The whole proteome of *C. reinhardtii* was used as a reference to demonstrate the presence of U amino acids

A contradictory result was also identified regarding the presence of selenocysteine amino acid encoding machinery in the fungal dataset. At least 134 fungal protein sequences from 112 fungal species were found with a functional annotation name associated with the term “selenocysteine” (Additional file 5). When the sequences of the 134 fungal proteins were analyzed to check the presence of a Sec (U) amino acid, none of the sequences were found to contain a U amino acid (Fig. 1).

Further, we extended our analysis to a few additional protein families that included terpene synthesis-related proteins and WRKY transcription factors. The analysis identified 374 fungal protein sequences which were annotated as “monoterpene/terpene synthase/indole-diterpene protein/sesquiterpene synthase” (Additional file 6). The protein sequences annotated as “monoterpene epsilon-lactone” (GA086201.1) were downloaded and used in a BLASTP search in The Arabidopsis Information Resources (TAIR) website (Lamesch et al. 2012), which did not result in the identification of any monoterpene or lactone genes or proteins (Additional file 8: Fig. 1). BLASTP analysis in the TAIR database using fungal proteins annotated as “terpene synthase” (accession number EIW83595.1) resulted in a BLASTP hit of an *Arabidopsis* gene/protein annotated as MYOSIN XI D rather than terpene synthase (Additional file 9: Fig. 2). Analysis of fungal proteins annotated as “sesquiterpene cyclase” (accession number XP_020126298.1) resulted in a BLASTP hit of an *Arabidopsis* radiation sensitive 23C DNA repair protein RAD23-3 rather than a sesquiterpene cyclase or related protein (Additional file 10: Fig. 3).

WRKY transcription factors regulate diverse cellular processes and are characterized by the presence of a conserved WRKY domain (Mohanta et al. 2016). Although several fungal protein sequences were annotated as “WRKY transcription factor” (accession number OAD77222.1, OAD77578.1, OAD78519.1, OAD78677.1 of *Phycomyces blakesleeanus*), none of them possessed a consensus WRKY amino acid domain (Additional file 7). Additionally, fungal proteins that were annotated as “leghemoglobin” (accession number KXX77420.1) were analyzed for their sequence similarity to leghemoglobin proteins in other species and for the presence of a globin domain. Results of the sequence similarity and motif scan (Additional file 11: Fig. 4) analysis did not identify any similarity to other leghemoglobin proteins.

## Discussion

CDPKs are characterized by the presence of an N-terminal domain, kinase domain, an auto-inhibitory domain, and a regulatory domain (Mohanta et al. 2015a,b). The regulatory domain is characterized by the presence of 4 calcium-binding EF-hands (Chandran et al. 2006; Asai et al. 2013). The EF-hands present in the regulatory domain of CDPKs are conserved and contain a D-x-D conserved amino acid sequence at the 14th and 16th position, which are responsible for binding Ca^2+^ ions (Mohanta et al. 2019a). In addition, CDPKs contain N-terminal palmitoylation and myristoylation sites (Mohanta et al. 2019a). Hence, it is important to identify the presence of all 4 domains, and N-terminal sequences that serve as palmitoylation and myristoylation sites, to annotate a protein as a CDPK. Notably, however, in our analysis of fungal proteins annotated as CDPKs, N-terminal sequences or regulatory domains were not found. This raises the question of how these fungal genes/proteins were annotated as CDPKs despite the complete absence of any calcium-binding EF-hand domain. Even homology-based annotation should not result in such a misleading identification when there is a complete lack of an EF-hand containing regulatory domain. Although CDPKs are commonly present in plant and animal species, it is well known that fungi do not encode any CDPKs in their genomes (Braun and Schulman 1995; Mohanta et al. 2015a, 2019a). Calcium-dependent protein kinases play diverse roles in plants and animals (Braun and Schulman 1995; Mohanta et al. 2015a, 2019a). In plants, CDPK regulates growth, development, and biotic and abiotic stress tolerance (Mohanta et al. 2012; Gao et al. 2014; Mohanta and Sinha 2016; Shi et al. 2018). In fungi, however, calcium-signaling events are regulated by calmodulins, calcineurin B-like proteins, calmodulin-like proteins, calcineurin-responsive zinc finger transcription factors, Ca^2+^ ATPase, Ca^2+^/H^+^ exchangers, a high-affinity calcium system, a low-affinity calcium system, transient receptor potential (TRP)-like calcium channels, and mitochondrial calcium channels (Tisi et al. 2016; Liu et al. 2015). Fungi do not utilize members of the *CDPK* gene family in calcium signaling processes, and do not encode CDPK genes in their genome. The basal calcium level in the cytoplasm of fungi ranges from 50 to 200 nM and fungi store the majority of their Ca^2+^ ions in the vacuole (approximately ~ 95%). Calmodulin, calcineurin B-like proteins, and other calcium-related proteins, maintain Ca^2+^ homeostasis without the utilization of CDPKs (Tisi et al. 2016). Cellular Ca^2+^-channels and cation/proton exchange channels regulate the growth in filamentous fungi and play a role in cell division, hyphal tip growth, and hyphal branching (Benčina et al. 2009). The vacuolar Ca^2+^-ATPase in fungi is closely related to a plasma membrane Ca^2+^-ATPase (PMCA)-type pump. PMCA contains a cytosolic auto-inhibitory domain at the C-terminal end, which is relieved by the binding of calmodulin (Tisi et al. 2016; Brini et al. 2013). The auto-inhibitory domain in PMCA proteins in fungi are functionally similar to the auto-inhibitory domain of CDPK proteins in plants and animals. The auto-inhibitory domain in PMCA proteins in fungi may represent a structural and functional equivalent of the auto-inhibitory domain found in CDPK proteins and regulate calcium-signaling events. The annotation error was not only found in the erroneous annotation of CDPKs, but also in the annotation of CaM and CML calcium binding proteins. CaM and CMLs are characterized by the presence of four calcium binding EF-hands (Mohanta et al. 2017). The fungal proteins annotated as CaM and CMLs, however, were found to possess two, three, or four calcium binding EF-hands, which again highlights the problem of erroneous annotations. Interestingly, only 14 CML proteins were identified among the 7.15 million analyzed fungal protein sequences, and only a few were found to possess four calcium binding EF-hands. The protein represented by accession GAO83098.1 (*Aspergillus udagawae*) contained only two EF-hands, while proteins with NCBI accession numbers KIY65364.1 (*Cylindrobasidium torrendii*) and RCH90477.1 (*Rhizopus azygosporus*) contained three EF-hands. When new proteins are annotated and added to a database, they should conform in sequence similarity and the presence of conserved domains previously established for a specific family of proteins. This condition appears to be completely lacking in many of the present annotations.

Selenoproteins contain Sec amino acid, which is encoded by the UGA codon. Proteins that are typically associated with the reactive oxygen species signaling machinery (glutathione peroxidase) contain Sec amino acid (Borchert et al. 2018). Animals (Labunskyy et al. 2014), plants (Mohanta et al. 2019b), and bacteria (Zhang et al. 2006) have all been reported to contain Sec amino acid. However, the presence of Sec amino acid has not been reported in the fungi (Mariotti and Guigó 2015). Previously, Mariotti and Guigó (2015) also reported that fungi do not contain Sec amino acid (Mariotti and Guigó 2015). Therefore, the presence of an ambiguous gene/protein annotation name with “selenocysteine” in the fungal genome/proteome is concerning for the research community. Therefore, it is important to provide an insight to the annotation strategy of the fungal genome, making it important for the researchers across the globe to consider it as a serious problem, and justifying the case to address this issue immediately. A previous study also reported the genome annotation error in bacteria (Brenner 1999; Devos and Valencia 2001). Several reasons may be accounted for the mis-annotation of the gene/protein sequences. However, the parameters for the placement of a lower or higher limit for the coding sequences can also attribute annotation errors. Another very probable reason may be the lack of a “gold standard” of reference sequences. However, when the mis-annotation occurs at the super-family level, that is CDPK, CaM, CMLs, and WRKY TFs, it is a concerning matter.

Terpene synthases represent a large family of protein encoding genes associated with the synthesis of diverse arrays of metabolites. Analysis of 689 fungal proteomes led us to find 374 protein sequences with annotation terms associated with “terpene”. Analysis of a few protein sequences led to a mismatch similarity result or the absence of a terpene synthase motif. Similarly, proteins with the annotation name “WRKY transcription factor” were found in the protein sequences of fungi (Additional file 7), but none of the proteins contained a WRKY domain. Furthermore, a protein with the annotation name “leghemoglobin” did not show the presence of globin motif and was still annotated as leghemoglobin. Leghemoglobin are the characteristic proteins of nitrogen fixing rhizobacteria which fix the atmospheric nitrogen during the symbiotic interaction with plants (Nadler and Avissar 1977). Although it is not impossible, the presence of leghemoglobin protein in fungi is highly unlikely and the absence of a globin domain in the analyzed protein provides further evidence of annotation errors.

A recent comparative study of protein-coding and lncRNA transcripts in the RefSeq and Gencode human gene databases found that only 27.5% of the Gencode transcripts had an exact match with the introns at the same position in the RefSeq database (Salzberg 2019). These data indicate that even after 19 years of continuous effort, the exon–intron boundary in genes within the human genome has not been completely resolved. The problems with yeast and *Arabidopsis* annotations are even greater than in the human genome (Salzberg 2019). Advances in RNA-sequencing technology may help to partially resolve such problems as a full-length transcript can be directly aligned to the genome to reveal exon–intron structure. The Mammalian Gene Collection, which includes the genes of humans and a few other species, could also help to reduce the error rate through an RNA-seq approach. The modern annotation pipeline, MAKER, uses RNA-seq data and aligns protein sequences found in other databases and provides correct annotations. Although RNA-seq has its own limitations, it remains a viable alternative to reduce and correct erroneous annotations. Errors in gene and genome assembly can also lead to annotation errors. Although automated genome annotation processes enable researchers to cope with the pace of sequencing large numbers of genomes, some of which may be very large in size, minor errors in existing annotations can directly propagate the error to the other species, thus amplifying the problem. Min et al. (2017) developed FunGAP, a fungal genome annotation pipeline that utilizes an evidence-based gene model evaluation for annotation (Min et al. 2017). This homology-based modeling approach uses domain homology to annotate a protein and represents a useful alternative for the annotation of fungal proteins.

## Conclusion

Homology/orthology-based sequence similarity using a high similarity value should be used as a “gold standard” to reduce and avoid annotation errors in the naming of fungal proteins. In addition, co-localization of functionally-linked genes, experimental proteomics and the presence of established motifs and conserved sequences would greatly contribute to error-free functional annotation of genes. The mitigation of false-positive and false-negative annotation errors that are being propagated on a regular basis can have a detrimental effect on the optimal and efficient use of database information, especially in the case of pathway analyses. Therefore, extra care and caution should be used when submitting the results of genome sequencing, assembly, and annotation.

## Methods

The annotated proteins from 689 fungal species were downloaded from the NCBI (National Center for Biotechnology Information) database, which provided approximately 7.15 million protein sequences. All of the annotated fungal proteins sequences available up to March 10, 2020 were downloaded. A simple search was made to identify protein sequences that were annotated as “selenocysteine/selenoprotein”. The resulting protein sequences were then analyzed for the presence of selenocysteine amino acids and the results were recorded. The protein sequences of the full proteome of *Chlamydomonas reinhardtii* were used as a reference to analyze for the presence and absence of Sec (U) amino acids. A similar analysis was conducted for the presence of CDPK proteins in fungi. Protein sequences annotated as “calcium dependent protein kinase” were subsequently analyzed on the ScanProsite website (Sigrist et al. 2012), employing a motif scan (https://myhits.sib.swiss/cgi-bin/motif_scan#), BLAST (Altschul et al. 1990; Johnson et al. 2008), and MEME suite (Bailey et al. 2009) using default parameters to determine the presence of calcium binding EF-hand domains. All of the analyses were conducted on a Linux-based platform.

## Supplementary Information

**Additional file 1.** Sequences annotated with the term calcium dependent protein kinase.

**Additional file 2.** Calmodulin proteins with four calcium binding EF-hands.

**Additional file 3.** Proteins annotated with the term “calmodulin” having two, or three calcium binding EF-hands instead of the characteristic four calcium binding EF-hands of calmodulin proteins.

**Additional file 4.** Fungal calmodulin-like (CML) proteins. CML proteins are assumed to contain four calcium binding EF-hands. The fungal CMLs, however, were found to contain two, three, or four calcium binding EF-hands.

**Additional file 5.** Fungal protein sequences annotated with the term “selenocysteine/selenoprotein”. Notably, none of the designated selenoprotein were found to possess any selenocysteine (U) amino acids.

**Additional file 6.** Fungal proteins annotated with terms related to the terpene synthesis pathway.

**Additional file 7.** Fungal proteins with conserved WRKY domains.

**Additional file 8: Fig. 1.** BLASTP analysis of fungal proteins annotated with the term monoterpene epsilon-lactone” (GA086201.1) in The Arabidopsis Information Resources (TAIR) database. BLASTP results did not identify any hits for terpene related proteins.

**Additional file 9: Fig. 2.** BLASTP analysis of fungal proteins annotated with the term “terpene synthase” (accession number EIW83595.1) in The Arabidopsis Information Resources (TAIR) database. Results identified a gene annotated as a Myosin XI D protein instead of a terpene synthase.

**Additional file 10: Fig. 3.** BLASTP analysis of fungal proteins annotated with the term “sesquiterpene cyclase” (accession number XP_020126298.1). BLASTP results identified a radiation sensitive 23C DNA repair protein (RAD23-3) rather than a sesquiterpene cyclase.

**Additional file 11: Fig. 4.** BLASTP analysis of a fungal protein annotated as a leghemoglobin (accession number KXX77420.1) protein against proteins in the NCBI database. Results only identified a hit to itself (KXX77420.1) and no other protein was found to be a leghemoglobin; indicating the erroneous annotation of fungal proteins.

## Data Availability

All the data associated with this study was taken from publicly available database and data associated with the manuscript is provided in supplementary files.

## Abbreviations

CDPK: Calcium dependent protein kinase
Sec: Selenocysteine
NCBI: National Center for Biotechnology Information
MEME: Multiple Em for motif elicitation
N-terminal: Amino terminal
EF-hand: Elongation factor hand
TRP: Transient receptor potential
PMCA: Plasma membrane Ca^2^^+^-ATPase
