# Supplementary material for: Fungal genomes: suffering with functional annotation errors
Source: IMA Fungus. 2021 Nov 1;12:32. doi: 10.1186/s43008-021-00083-x (PMC8559351; doi:10.1186/s43008-021-00083-x)
Supplement: Supplementary file 8 — Additional file 8: Fig. 1. BLASTP analysis of fungal proteins annotated with the term monoterpene epsilon-lactone” (GA086201.1) in The Arabidopsis Information Resources (TAIR) database. BLASTP results did not identify any hits for terpene related proteins. [file 43008_2021_83_MOESM8_ESM.pdf]

blastp query on Araport11 protein sequences (protein) sequences

Query performed by the The Arabidopsis Information Resource (TAIR); for full BLAST options and parameters, refer to the NCBI BLAST Documentation

Your comments and suggestions are requested: Send a Message to TAIR

Summary of BLAST Results Help

The graph shows the highest hits per range.
Data have been omitted in the Summary Graphic: 23/28 hits displayed.

| Symbols:no symbol available | no full name available | Chr2:1077080-1078018 FORWARD LENGTH=312

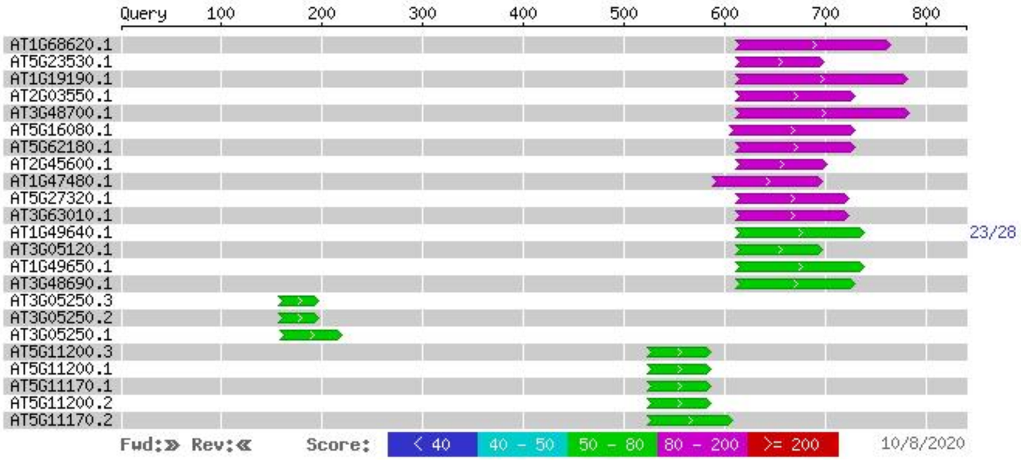


```
BLASTP 2.9.0+


Reference:
Stephen F. Altschul, Thomas L. Madden, Alejandro A. Schäffer,
Jinghui Zhang, Zheng Zhang, Webb Miller, and David J. Lipman (1997),
"Gapped BLAST and PSI-BLAST: a new generation of protein database
search programs", Nucleic Acids Res. 25:3389-3402.


Reference for
composition-based statistics:
Alejandro A. Schäffer, L. Aravind, Thomas L. Madden, Sergei
Shavirin, John L. Spouge, Yuri I. Wolf, Eugene V. Koonin, and
Stephen F. Altschul (2001), "Improving the accuracy of PSI-BLAST
protein database searches with composition-based statistics and
other refinements", Nucleic Acids Res. 29:2994-3005.



Database:  Araport11 protein sequences (protein)
           48,359 sequences; 20,855,782 total letters



Query= user-submitted sequence

Length=840
                                                                      Score        E
```

```
Sequences producing significant alignments:                      (Bits)     Value

AT1G68620.1 | Symbols:no symbol available | no full name availabl...  54.7       2e-07
AT5G23530.1 | Symbols:CXE18,AtCXE18 | carboxyesterase 18 | Chr5:7...  52.8       7e-07
AT1G19190.1 | Symbols:no symbol available | no full name availabl...  46.6       6e-05
AT2G03550.1 | Symbols:no symbol available | no full name availabl...  45.1       2e-04
AT3G48700.1 | Symbols:ATCXE13,CXE13 | carboxyesterase 13 | Chr3:1...  45.1       2e-04
AT5G16080.1 | Symbols:AtCXE17,CXE17 | carboxyesterase 17 | Chr5:5...  42.0       0.002
AT5G62180.1 | Symbols:CXE20,AtCXE20 | carboxyesterase 20 | Chr5:2...  41.2       0.003
AT2G45600.1 | Symbols:no symbol available | no full name availabl...  40.0       0.006
AT1G47480.1 | Symbols:no symbol available | no full name availabl...  38.1       0.028
AT5G27320.1 | Symbols:ATGID1C,GID1C | GA INSENSITIVE DWARF1C | Ch...  36.6       0.098
AT3G63010.1 | Symbols:ATGID1B,GID1B | GA INSENSITIVE DWARF1B | Ch...  35.8       0.18
AT1G49640.1 | Symbols:no symbol available | no full name availabl...  34.3       0.45
AT3G05120.1 | Symbols:ATGID1A,GID1A | GA INSENSITIVE DWARF1A | Ch...  34.3       0.48
AT1G49650.1 | Symbols:no symbol available | no full name availabl...  33.5       0.83
AT3G48690.1 | Symbols:ATCXE12,CXE12 | ARABIDOPSIS THALIANA CARBOX...  33.1       0.96
AT3G05250.3 | Symbols:no symbol available | no full name availabl...  32.0       2.1
AT3G05250.2 | Symbols:no symbol available | no full name availabl...  32.0       2.1
AT3G05250.1 | Symbols:no symbol available | no full name availabl...  31.6       3.0
AT5G11200.3 | Symbols:UAP56b | homolog of human UAP56 b | Chr5:35...  31.6       4.2
AT5G11200.1 | Symbols:UAP56b | homolog of human UAP56 b | Chr5:35...  31.2       5.2
AT5G11170.1 | Symbols:UAP56a | homolog of human UAP56 a | Chr5:35...  31.2       5.2
AT5G11200.2 | Symbols:UAP56b | homolog of human UAP56 b | Chr5:35...  30.8       5.8
AT5G11170.2 | Symbols:UAP56a | homolog of human UAP56 a | Chr5:35...  30.8       6.5
AT2G28470.3 | Symbols:BGAL8 | beta-galactosidase 8 | Chr2:1216904...  30.8       7.3
AT2G28470.1 | Symbols:BGAL8 | beta-galactosidase 8 | Chr2:1216904...  30.8       7.3
AT2G28470.4 | Symbols:BGAL8 | beta-galactosidase 8 | Chr2:1216904...  30.8       7.4
AT2G28470.2 | Symbols:BGAL8 | beta-galactosidase 8 | Chr2:1216904...  30.8       7.4
AT5G14310.1 | Symbols:AtCXE16,CXE16 | carboxyesterase 16 | Chr5:4...  30.4       8.3


>AT1G68620.1 | Symbols:no symbol available | no full name available |
Chr1:25766018-25767028
FORWARD LENGTH=336
Length=336

 Score = 54.7 bits (130),  Expect = 2e-07, Method: Compositional matrix adjust.
 Identities = 49/167 (29%), Positives = 70/167 (42%), Gaps = 32/167 (19%)

Query  611  IVINFHGGGFAMDATA--PYLDFWPRIQKSLASADIETAWFHCTYTLTPHAAYPTQFEEA  668
            +++ FHGGGF + + +   Y +F  R+     SA          Y L P    P  +E+
Sbjct  91   LIVYFHGGGFCVGSASWLCYHEFLARL-----SARSRCLVMSVNYRLAPENPLPAAYEDG  145

Query  669  VEALRYIVE-----------DLGRSPSQILLAGDSAGANLCLAVLSHLTHPSDGVPELII  717
            V A+ ++ +           D GR    I LAGDSAG N+   V + L  P D    L +
Sbjct  146  VNAILWLNKARNDNLWAKQCDFGR----IFLAGDSAGGNIAQQVAARLASPED----LAL  197

Query  718  KEPIRGVILMSPDIDAREVMTEWSRAYPNGWPSNNYIEAAEASEGWW  764
            K  I G IL+ P     E  TE  R   N       +    +S+ WW
Sbjct  198  K--IEGTILIQPFYSGEE-RTESERRVGN---DKTAVLTLASSDAWW  238


>AT5G23530.1 | Symbols:CXE18,AtCXE18 | carboxyesterase 18 |  Chr5:7933366-7934373
REVERSE LENGTH=335
Length=335

 Score = 52.8 bits (125),  Expect = 7e-07, Method: Compositional matrix adjust.
 Identities = 33/96 (34%), Positives = 47/96 (49%), Gaps = 13/96 (14%)

Query  611  IVINFHGGGFAMDA--TAPYLDFWPRIQKSLASADIETAWFHCTYTLTPHAAYPTQFEEA  668
            +V+ FHGGGFA  +    PY +   R  + L +  I        Y L P   YP Q+++
Sbjct  89   VVVFFHGGGFAFLSPNAYPYDNVCRRFARKLPAYVISV-----NYRLAPEHRYPAQYDDG  143

Query  669  VEALRYIVEDLGR------SPSQILLAGDSAGANLC  698
             +AL+YI E+ G         S+   AGDSAG N+
Sbjct  144  FDALKYIEENHGSILPANADLSRCFFAGDSAGGNIA  179
```

```
>AT1G19190.1 | Symbols:no symbol available | no full name available |
Chr1:6623876-6624832
FORWARD LENGTH=318
Length=318

 Score = 46.6 bits (109),  Expect = 6e-05, Method: Compositional matrix adjust.
 Identities = 50/196 (26%), Positives = 82/196 (42%), Gaps = 33/196 (17%)

Query  611  IVINFHGGGFAMDATAPYLDFWPRIQKSLASADIETAWFHCT--YTLTPHAAYPTQFEEA  668
            +++ FHGGGF M+       F P     L SA   T     +  Y   P    PT +E++
Sbjct  74   LLVYFHGGGFIMETA-----FSPIYHTFLTSAVSATDCIAVSVEYRRAPEHPIPTLYEDS  128

Query  669  VEALRYIVEDLGRSP----------SQILLAGDSAGANLCLAVLSHLTHPSDGVPELII  717
             +A+++I   + RS            S++ LAGDSAGAN+   +   +    + +P
Sbjct  129  WDAIQWIFTHITRSGPEDWLNKHADFSKVFLAGDSAGANIAHHMAIRVD--KEKLPPENF  186

Query  718  KEPIRGVILMSPDIDAR----EVMTEWSRAYPNGWPSNNYIEAAEASEGWWN-------N  766
            K  I G+IL  P   ++     E+  E  R Y   W   +        + W N
Sbjct  187  K--ISGMILFHPYFLSKALIEEMEVEAMRYYERLWRIASPDSGNGVEDPWINVVGSDLTG  244

Query  767  TQVQQILVLAGGDEAL  782
               +++LV+  G++ L
Sbjct  245  LGCRRVLVMVAGNDVL  260


>AT2G03550.1 | Symbols:no symbol available | no full name available |
Chr2:1077080-1078018
FORWARD LENGTH=312
Length=312

 Score = 45.1 bits (105),  Expect = 2e-04, Method: Compositional matrix adjust.
 Identities = 37/132 (28%), Positives = 63/132 (48%), Gaps = 24/132 (18%)

Query  611  IVINFHGGGFAMDA--TAPYLDFWPRIQKSLASADIETAWFHCTYTLTPHAAYPTQFEEA  668
            I+I FHGGGF ++    + PY  F   +  ++A+A+         Y   P    P  +E++
Sbjct  70   ILIYFHGGGFIIETAFSPPYHTF---LTSAVAAANCLA--ISVNYRRAPEFPVPIPYEDS  124

Query  669  VEALRYIVEDL-GRSP----------SQILLAGDSAGANLCLAVLSHLTHPSDGVPELII  717
             ++L++++  + G  P            ++ LAGDSAG N+      HLT  +    E +
Sbjct  125  WDSLKWVLTHITGTGPETWINKHGDFGKVFLAGDSAGGNIS----HHLTMRAK--KEKLC  178

Query  718  KEPIRGVILMSP  729
               I G+IL+ P
Sbjct  179  DSLISGIILIHP  190


>AT3G48700.1 | Symbols:ATCXE13,CXE13 | carboxyesterase 13 |  Chr3:18038825-18039814
REVERSE LENGTH=329
Length=329

 Score = 45.1 bits (105),  Expect = 2e-04, Method: Compositional matrix adjust.
 Identities = 46/204 (23%), Positives = 91/204 (45%), Gaps = 38/204 (19%)

Query  611  IVINFHGGGFAMDATAPYLDFWPRIQKSLASADIETAWFHCTYTLTPHAAYPTQFEEAVE  670
            +++ FHGGGF ++ TA    +   +++++D         Y   P    PT ++++
Sbjct  76   LLVYFHGGGFLVE-TAFSPTYHTFLTAAVSASDCVAV--SVDYRRAPEHPIPTSYDDSWT  132

Query  671  ALRYIVEDLGRSPSQ-----------ILLAGDSAGANLCLAVLSHLTHPSDG---VPELI  716
            AL+++   +  S S+           + LAGDSAGAN+      H+T  +      PE +
Sbjct  133  ALKWVFSHIAGSGSEDWLNKHADFSKVFLAGDSAGANIT----HHMTMKAAKDKLSPESL  188

Query  717  IKEPIRGVILMSP------DIDARE---------VMTEWSRAYPNGWPSNN--YIEAAEA  759
             +  I G+IL+ P       +D +E          + + W+ A PN    ++  +I   ++
Sbjct  189  NESGISGIILVHPYFWSKTPVDDKETTDVAIRTWIESVWTLASPNSKDGSDDPFINVVQS  248

Query  760  SEGWWNNTQVQQILVLAGGDEALL  783
                +     ++LV+    +AL+
Sbjct  249  ESVDLSGLGCGKVLVMVAEKDALV  272
```

```
>AT5G16080.1 | Symbols:AtCXE17,CXE17 | carboxyesterase 17 |  Chr5:5252533-5253567
REVERSE LENGTH=344
Length=344

 Score = 42.0 bits (97),  Expect = 0.002, Method: Compositional matrix adjust.
 Identities = 38/141 (27%), Positives = 58/141 (41%), Gaps = 24/141 (17%)

Query  604  GSPSARY-IVINFHGGGFAMDATA--PYLDFWPRIQKSLASADIETAWFHCTYTLTPHAA  660
             SPS    +++ FHGGGF + + A   Y DF   +        +        Y L P
Sbjct  86   ASPSVTLPLLVYFHGGGFCVGSAAWSCYHDFLTSLVKARCVIVSV-----NYRLAPEHR  140

Query  661  YPTQFEEAVEALRYIVEDL----GRSPS--------QILLAGDSAGANLCLAVLSHLTHP  708
             P  +++ V  + ++V+      G  PS         + LAGDSAGAN+   V   +
Sbjct  141  LPAAYDDGVNVVSWLVKQQISTGGGYPSWLSKCNLSNVFLAGDSAGANIAYQVAVRIMAS  200

Query  709  SDGVPELIIKEPIRGVILMSP  729
                   L +K    G+IL+ P
Sbjct  201  GKYANTLHLK----GIILIHP  217


>AT5G62180.1 | Symbols:CXE20,AtCXE20 | carboxyesterase 20 |  Chr5:24978866-24979849
REVERSE LENGTH=327
Length=327

 Score = 41.2 bits (95),  Expect = 0.003, Method: Compositional matrix adjust.
 Identities = 39/128 (30%), Positives = 54/128 (42%), Gaps = 19/128 (15%)

Query  611  IVINFHGGGFAMDATAPYL--DFWPRIQKSLASADIETAWFHCTYTLTPHAAYPTQFEEA  668
            IV+ +HGGGF + +    L  DF   + +     D+       +Y L P    P  +++
Sbjct  82   IVVYYHGGGFILCSVDMQLFHDFCSEVAR-----DLNAIVVSPSYRLAPEHRLPAAYDDG  136

Query  669  VEALRYIVEDLGR------SPSQILLAGDSAGANLCLAV-LSHLTHPSDGVPELIIKEPI  721
            VEAL +I              S + L G SAG NL   V L  +   SD  P       I
Sbjct  137  VEALDWIKTSDDEWIKSHADFSNVFLMGTSAGGNLAYNVGLRSVDSVSDLSP-----LQI  191

Query  722  RGVILMSP  729
            RG+IL  P
Sbjct  192  RGLILHHP  199


>AT2G45600.1 | Symbols:no symbol available | no full name available |
Chr2:18789799-18790788
FORWARD LENGTH=329
Length=329

 Score = 40.0 bits (92),  Expect = 0.006, Method: Compositional matrix adjust.
 Identities = 30/109 (28%), Positives = 48/109 (44%), Gaps = 24/109 (22%)

Query  611  IVINFHGGGFAM--DATAPYLDFWPRIQKSLASADIETAWFHCTYTLTPHAAYPTQFEEA  668
            I++ FHGGGF +   A+AP+ +   ++   L     +T      Y L P    P  +E+A
Sbjct  68   ILVYFHGGGFILYSAASAPFHESCTKMADRL-----QTIILSVEYRLAPEHRLPAAYEDA  122

Query  669  VEALRYIVEDLGRSP----------------SQILLAGDSAGANLCLAV  701
            VEA+ ++  D  R P                S+  + G S+G N+   V
Sbjct  123  VEAILWL-RDQARGPINGGDCDTWLKDGVDFSKCYVMGSSSGGNIVYNV  170


>AT1G47480.1 | Symbols:no symbol available | no full name available |
Chr1:17417623-17419296
FORWARD LENGTH=314
Length=314

 Score = 38.1 bits (87),  Expect = 0.028, Method: Compositional matrix adjust.
 Identities = 31/118 (26%), Positives = 54/118 (46%), Gaps = 11/118 (9%)

Query  587  LRPHFVSTSRGLKGFWIGSPSARYIVINFHGGGFAMDATAPYLDFWPRIQKSLASADIET  646
            + P    ++R  + F I       +++ FHGG F + +T+ +  +    + K +  A++
Sbjct  50   IEPKTGLSARIYRPFSIQPGQKIPLMLYFHGGAFLISSTS-FPSYHTSLNKIVNQANVIA  108
```

```
Query  647  AWFHCTYTLTPHAAYPTQFEEAVEALRYI-------VEDLGRSPSQILLAGDSAGANL  697
                  Y L P    PT +E++  AL+ I       + D      S + L GDSAGAN+
Sbjct  109  V--SVNYRLAPEHPLPTAYEDSWTALKNIQAINEPWINDYADLDS-LFLVGDSAGANI  163


>AT5G27320.1 | Symbols:ATGID1C,GID1C | GA INSENSITIVE DWARF1C |  Chr5:9629254-9630746
FORWARD LENGTH=344
Length=344

 Score = 36.6 bits (83),  Expect = 0.098, Method: Compositional matrix adjust.
 Identities = 30/119 (25%), Positives = 48/119 (40%), Gaps = 10/119 (8%)

Query  611  IVINFHGGGFA-MDATAPYLDFWPRIQKSLASADIETAWFHCTYTLTPHAAYPTQFEEAV  669
            +++ FHGG FA   A +   D   R    L  A + +      Y   P   YP  +++
Sbjct  106  VIVFFHGGSFAHSSANSAIYDTLCRRLVGLCGAVVVSV----NYRRAPENRYPCAYDDGW  161

Query  670  EALRYI-----VEDLGRSPSQILLAGDSAGANLCLAVLSHLTHPSDGVPELIIKEPIRG  723
              L+++     +     S  +I LAGDS+G N+   V          V   I+  P+ G
Sbjct  162  AVLKWVNSSSWLRSKKDSKVRIFLAGDSSGGNIVHNVAVRAVESRIDVLGNILLNPMFG  220


>AT3G63010.1 | Symbols:ATGID1B,GID1B | GA INSENSITIVE DWARF1B |
Chr3:23289717-23290998
FORWARD LENGTH=358
Length=358

 Score = 35.8 bits (81),  Expect = 0.18, Method: Compositional matrix adjust.
 Identities = 30/120 (25%), Positives = 50/120 (42%), Gaps = 12/120 (10%)

Query  611  IVINFHGGGFAMDA--TAPYLDFWPRIQKSLASADIETAWFHCTYTLTPHAAYPTQFEEA  668
            ++I FHGG F   +  +A Y  F  R+        +        Y  +P   YP  +++
Sbjct  108  VLIFFHGGSFTHSSANSAIYDTFCRRLVTICGVVVVSV-----DYRRSPEHRYPCAYDDG  162

Query  669  VEALRYI-----VEDLGRSPSQILLAGDSAGANLCLAVLSHLTHPSDGVPELIIKEPIRG  723
              AL ++     ++    S   + LAGDS+G N+   V    T+    V   I+  P+ G
Sbjct  163  WNALNWVKSRVWLQSGKDSNVYVYLAGDSSGGNIAHNVAVRATNEGVKVLGNILLHPMFG  222


>AT1G49640.1 | Symbols:no symbol available | no full name available |
Chr1:18375697-18376644
REVERSE LENGTH=315
Length=315

 Score = 34.3 bits (77),  Expect = 0.45, Method: Compositional matrix adjust.
 Identities = 34/141 (24%), Positives = 58/141 (41%), Gaps = 28/141 (20%)

Query  611  IVINFHGGGFAMDATAPYLDFWPRIQKSLASADIETAWFHCT--YTLTPHAAYPTQFEEA  668
            ++I FHGG + + +      F P     L    I       +  Y L P    P  ++++
Sbjct  76   LLIYFHGGAYIIQSP-----FSPVYHNYLTEVVITANCLAVSVQYRLAPEHPVPAAYDDS  130

Query  669  VEALRYIVE----------DLGRSPSQILLAGDSAGANLCLAVLSHLTHPSDGVPELIIK  718
              A+++I              D  R     + +AGDSAGAN     +SH      G  +L
Sbjct  131  WSAIQWIFSHSDDWINEYADFDR----VFIAGDSAGAN-----ISHHMGIRAGKEKL--S  179

Query  719  EPIRGVILMSPDIDAREVMTE  739
              I+G++++ P    +E + E
Sbjct  180  PTIKGIVMVHPGFWGKEPIDE  200


>AT3G05120.1 | Symbols:ATGID1A,GID1A | GA INSENSITIVE DWARF1A |  Chr3:1430682-1432287
FORWARD LENGTH=345
Length=345

 Score = 34.3 bits (77),  Expect = 0.48, Method: Compositional matrix adjust.
 Identities = 25/93 (27%), Positives = 40/93 (43%), Gaps = 10/93 (11%)

Query  611  IVINFHGGGFA-MDATAPYLDFWPRIQKSLASADIETAWFHCTYTLTPHAAYPTQFEEAV  669
            +++ FHGG FA   A +   D   R    L    + +      Y   P   YP  +++
```

```
Sbjct  108  VILFFHGGSFAHSSANSAIYDTLCRRLVGLCKCVVVSV----NYRRAPENPYPCAYDDGW  163

Query  670  EALRYI-----VEDLGRSPSQILLAGDSAGANL  697
             AL ++     ++    S   I LAGDS+G N+
Sbjct  164  IALNWVNSRSWLKSKKDSKVHIFLAGDSSGGNI  196


>AT1G49650.1 | Symbols:no symbol available | no full name available |
Chr1:18377363-18378487
REVERSE LENGTH=374
Length=374

 Score = 33.5 bits (75),  Expect = 0.83, Method: Compositional matrix adjust.
 Identities = 34/142 (24%), Positives = 59/142 (42%), Gaps = 25/142 (18%)

Query  611  IVINFHGGGFAMDA--TAPYLDFWPRIQKSLASADIETAWFHCTYTLTPHAAYPTQFEEA  668
            ++I FHGG +  ++  +  Y +F   + KS     +        Y   P    P  +E+
Sbjct  130  LLIYFHGGAWINESPFSPIYHNFLTEVVKSANCLAVSV-----QYRRAPEDPVPAAYEDT  184

Query  669  VEALRYIVEDLGRSPSQ-----------ILLAGDSAGANLCLAVLSHLTHPSDGVPELII  717
              A+++I      S  +           + LAGDSAG N     +SH      G  +L
Sbjct  185  WSAIQWIFSHSCGSGEEDWINKYADFERVFLAGDSAGGN-----ISHHMAMRAGKEKL--  237

Query  718  KEPIRGVILMSPDIDAREVMTE  739
            K  I+G +++ P I  ++ + E
Sbjct  238  KPRIKGTVIVHPAIWGKDPVDE  259


>AT3G48690.1 | Symbols:ATCXE12,CXE12 | ARABIDOPSIS THALIANA CARBOXYESTERASE
12 |  Chr3:18037186-18038160 REVERSE LENGTH=324
Length=324

 Score = 33.1 bits (74),  Expect = 0.96, Method: Compositional matrix adjust.
 Identities = 32/132 (24%), Positives = 62/132 (47%), Gaps = 20/132 (15%)

Query  611  IVINFHGGGFAMDATAPYLDFWPRIQKSLASADIETAWFHCTYTLTPHAAYPTQFEEAVE  670
            +++ FHGGGF ++ TA    +   +  S+++++         Y   P       F+++
Sbjct  73   LLVYFHGGGFIIE-TAFSPTYHTFLTTSVSASNCVAV--SVDYRRAPEHPISVPFDDSWT  129

Query  671  ALRYIVEDLGRSP-----------SQILLAGDSAGANLC--LAVLSHLTHPSDGVPELII  717
            AL+++   +  S            S++ L+GDSAGAN+   +A+ +     S G+ +
Sbjct  130  ALKWVFTHITGSGQEDWLNKHADFSRVFLSGDSAGANIVHHMAMRAAKEKLSPGLND---  186

Query  718  KEPIRGVILMSP  729
               I G+IL+ P
Sbjct  187  -TGISGIILLHP  197


>AT3G05250.3 | Symbols:no symbol available | no full name available |
Chr3:1496207-1497387
FORWARD LENGTH=209
Length=209

 Score = 32.0 bits (71),  Expect = 2.1, Method: Compositional matrix adjust.
 Identities = 15/42 (36%), Positives = 26/42 (62%), Gaps = 1/42 (2%)

Query  156  SAFVRESDVDVLRHRIRPLIEDVCNKIKD-KKPESLLAKTWN  196
             A ++E DVD++ H +  ++E  C +IK  +K E+  A+T N
Sbjct  81   QALMQEEDVDIVLHHLVGVMESFCKRIKQRRKQETRSAETTN  122


>AT3G05250.2 | Symbols:no symbol available | no full name available |
Chr3:1496207-1497387
FORWARD LENGTH=209
Length=209

 Score = 32.0 bits (71),  Expect = 2.1, Method: Compositional matrix adjust.
 Identities = 15/42 (36%), Positives = 26/42 (62%), Gaps = 1/42 (2%)
```

```
Query  156  SAFVRESDVDVLRHRIRPLIEDVCNKIKD-KKPESLLAKTWN  196
             A ++E DVD++ H +  ++E  C +IK  +K E+  A+T N
Sbjct  81   QALMQEEDVDIVLHHLVGVMESFCKRIKQRRKQETRSAETTN  122


>AT3G05250.1 | Symbols:no symbol available | no full name available |
Chr3:1495635-1497387
FORWARD LENGTH=278
Length=278

 Score = 31.6 bits (70),  Expect = 3.0, Method: Compositional matrix adjust.
 Identities = 18/64 (28%), Positives = 36/64 (56%), Gaps = 3/64 (5%)

Query  157  AFVRESDVDVLRHRIRPLIEDVCNKIKD-KKPESLLAKTWNFGRHSLRVVLERSSWDRLL  215
            A ++E DVD++ H +  ++E  C +IK  +K E+  A+T N  +   + V+  ++   ++
Sbjct  151  ALMQEEDVDIVLHHLVGVMESFCKRIKQRRKQETRSAETTN--QEQFKAVVSEAARPFVM  208

Query  216  AVVD  219
            A  D
Sbjct  209  ARTD  212


>AT5G11200.3 | Symbols:UAP56b | homolog of human UAP56 b |  Chr5:3567389-3570686
FORWARD LENGTH=468
Length=468

 Score = 31.6 bits (70),  Expect = 4.2, Method: Compositional matrix adjust.
 Identities = 21/65 (32%), Positives = 28/65 (43%), Gaps = 1/65 (2%)

Query  522  CISGGMSGKERYVEWKRVPMAHEEISSAATAVSTTKIEKRIQLTENRYMFKSYDQLYLHY  581
            CI  GMS +ER   +K     H+ I  A   V      +R+ +  N  M  S D  YLH
Sbjct  358  CIHSGMSQEERLTRYKSFKEGHKRILVATDLVGRGIDIERVNIVINYDMPDSADT-YLHR  416

Query  582  CQKNG  586
              + G
Sbjct  417  VGRAG  421


>AT5G11200.1 | Symbols:UAP56b | homolog of human UAP56 b |  Chr5:3567389-3570686
FORWARD LENGTH=427
Length=427

 Score = 31.2 bits (69),  Expect = 5.2, Method: Compositional matrix adjust.
 Identities = 21/65 (32%), Positives = 28/65 (43%), Gaps = 1/65 (2%)

Query  522  CISGGMSGKERYVEWKRVPMAHEEISSAATAVSTTKIEKRIQLTENRYMFKSYDQLYLHY  581
            CI  GMS +ER   +K     H+ I  A   V      +R+ +  N  M  S D  YLH
Sbjct  317  CIHSGMSQEERLTRYKSFKEGHKRILVATDLVGRGIDIERVNIVINYDMPDSADT-YLHR  375

Query  582  CQKNG  586
              + G
Sbjct  376  VGRAG  380


>AT5G11170.1 | Symbols:UAP56a | homolog of human UAP56 a |  Chr5:3553334-3556646
FORWARD LENGTH=427
Length=427

 Score = 31.2 bits (69),  Expect = 5.2, Method: Compositional matrix adjust.
 Identities = 21/65 (32%), Positives = 28/65 (43%), Gaps = 1/65 (2%)

Query  522  CISGGMSGKERYVEWKRVPMAHEEISSAATAVSTTKIEKRIQLTENRYMFKSYDQLYLHY  581
            CI  GMS +ER   +K     H+ I  A   V      +R+ +  N  M  S D  YLH
Sbjct  317  CIHSGMSQEERLTRYKSFKEGHKRILVATDLVGRGIDIERVNIVINYDMPDSADT-YLHR  375

Query  582  CQKNG  586
              + G
Sbjct  376  VGRAG  380
```

```
>AT5G11200.2 | Symbols:UAP56b | homolog of human UAP56 b |  Chr5:3567389-3570686
FORWARD LENGTH=486
Length=486

 Score = 30.8 bits (68),  Expect = 5.8, Method: Compositional matrix adjust.
 Identities = 21/65 (32%), Positives = 28/65 (43%), Gaps = 1/65 (2%)

Query  522  CISGGMSGKERYVEWKRVPMAHEEISSAATAVSTTKIEKRIQLTENRYMFKSYDQLYLHY  581
            CI  GMS +ER   +K     H+ I  A   V      +R+ +  N  M  S D  YLH
Sbjct  376  CIHSGMSQEERLTRYKSFKEGHKRILVATDLVGRGIDIERVNIVINYDMPDSADT-YLHR  434

Query  582  CQKNG  586
              + G
Sbjct  435  VGRAG  439


>AT5G11170.2 | Symbols:UAP56a | homolog of human UAP56 a |  Chr5:3554272-3556646
FORWARD LENGTH=344
Length=344

 Score = 30.8 bits (68),  Expect = 6.5, Method: Compositional matrix adjust.
 Identities = 26/86 (30%), Positives = 37/86 (43%), Gaps = 6/86 (7%)

Query  522  CISGGMSGKERYVEWKRVPMAHEEISSAATAVSTTKIEKRIQLTENRYMFKSYDQLYLHY  581
            CI  GMS +ER   +K     H+ I  A   V      +R+ +  N  M  S D  YLH
Sbjct  234  CIHSGMSQEERLTRYKSFKEGHKRILVATDLVGRGIDIERVNIVINYDMPDSADT-YLHR  292

Query  582  CQKNGLRPHFVSTSRGLKGFWIGSPS  607
              + G    F   ++GL   ++ S S
Sbjct  293  VGRAG---RF--GTKGLAITFVASAS  313


>AT2G28470.3 | Symbols:BGAL8 | beta-galactosidase 8 |  Chr2:12169047-12173164
REVERSE LENGTH=852
Length=852

 Score = 30.8 bits (68),  Expect = 7.3, Method: Compositional matrix adjust.
 Identities = 14/34 (41%), Positives = 20/34 (59%), Gaps = 2/34 (6%)

Query  675  IVEDLGRSPSQILLAGDSAGANLCLAVLSHLTHP  708
            + E++G  P+QI  A    G+NLCL V    +HP
Sbjct  719  LFEEMGGDPTQISFATKQTGSNLCLTV--SQSHP  750


>AT2G28470.1 | Symbols:BGAL8 | beta-galactosidase 8 |  Chr2:12169047-12173164
REVERSE LENGTH=852
Length=852

 Score = 30.8 bits (68),  Expect = 7.3, Method: Compositional matrix adjust.
 Identities = 14/34 (41%), Positives = 20/34 (59%), Gaps = 2/34 (6%)

Query  675  IVEDLGRSPSQILLAGDSAGANLCLAVLSHLTHP  708
            + E++G  P+QI  A    G+NLCL V    +HP
Sbjct  719  LFEEMGGDPTQISFATKQTGSNLCLTV--SQSHP  750


>AT2G28470.4 | Symbols:BGAL8 | beta-galactosidase 8 |  Chr2:12169047-12173146
REVERSE LENGTH=846
Length=846

 Score = 30.8 bits (68),  Expect = 7.4, Method: Compositional matrix adjust.
 Identities = 14/34 (41%), Positives = 20/34 (59%), Gaps = 2/34 (6%)

Query  675  IVEDLGRSPSQILLAGDSAGANLCLAVLSHLTHP  708
            + E++G  P+QI  A    G+NLCL V    +HP
Sbjct  713  LFEEMGGDPTQISFATKQTGSNLCLTV--SQSHP  744
```

```
>AT2G28470.2 | Symbols:BGAL8 | beta-galactosidase 8 |  Chr2:12169047-12173146
REVERSE LENGTH=846
Length=846

 Score = 30.8 bits (68),  Expect = 7.4, Method: Compositional matrix adjust.
 Identities = 14/34 (41%), Positives = 20/34 (59%), Gaps = 2/34 (6%)

Query  675  IVEDLGRSPSQILLAGDSAGANLCLAVLSHLTHP  708
            + E++G  P+QI  A    G+NLCL V    +HP
Sbjct  713  LFEEMGGDPTQISFATKQTGSNLCLTV--SQSHP  744


>AT5G14310.1 | Symbols:AtCXE16,CXE16 | carboxyesterase 16 |  Chr5:4615592-4617422
FORWARD LENGTH=446
Length=446

 Score = 30.4 bits (67),  Expect = 8.3, Method: Compositional matrix adjust.
 Identities = 18/67 (27%), Positives = 30/67 (45%), Gaps = 7/67 (10%)

Query  611  IVINFHGGGFAMDATAPYLD--FWPRIQKSLASADIETAWFHCTYTLTPHAAYPTQFEEA  668
            +++ FHGGG+   ++    +  F  RI K       +       Y L P   YP  FE+
Sbjct  153  VMLQFHGGGWVSGSSDSAANDFFCRRIAKV-----CDVIVLAVGYRLAPENRYPAAFEDG  207

Query  669  VEALRYI  675
            V+ L ++
Sbjct  208  VKVLHWL  214



Lambda      K        H        a         alpha
   0.321    0.137    0.433    0.792     4.96

Gapped
Lambda      K        H        a         alpha    sigma
   0.267   0.0410    0.140     1.90     42.6     43.6

Effective search space used: 11290703157


  Database:  Araport11 protein sequences (protein)
    Posted date:  May 5, 2018  8:08 AM
  Number of letters in database: 20,855,782
  Number of sequences in database:  48,359



Matrix: BLOSUM62
Gap Penalties: Existence: 11, Extension: 1
Neighboring words threshold: 11
Window for multiple hits: 40
```

printer-friendly version

General comments or questions: curator@arabidopsis.org
Seed or DNA stock questions (donations, availability, orders, etc):abrc@osu.edu
