# Supplementary material for: Fungal genomes: suffering with functional annotation errors
Source: IMA Fungus. 2021 Nov 1;12:32. doi: 10.1186/s43008-021-00083-x (PMC8559351; doi:10.1186/s43008-021-00083-x)
Supplement: Supplementary file 9 — Additional file 9: Fig. 2. BLASTP analysis of fungal proteins annotated with the term “terpene synthase” (accession number EIW83595.1) in The Arabidopsis Information Resources (TAIR) database. Results identified a gene annotated as a Myosin XI D protein instead of a terpene synthase. [file 43008_2021_83_MOESM9_ESM.pdf]

blastp query on Araport11 protein sequences (protein) sequences

Query performed by the The Arabidopsis Information Resource (TAIR); for full BLAST options and parameters, refer to the NCBI BLAST Documentation

Your comments and suggestions are requested: Send a Message to TAIR

Summary of BLAST Results Help

All hits shown.

| Symbols:ATXID,XID | MYOSIN XI D,myosin XI D | Chr2:14086942-14096914 REVERSE LENGTH=1757

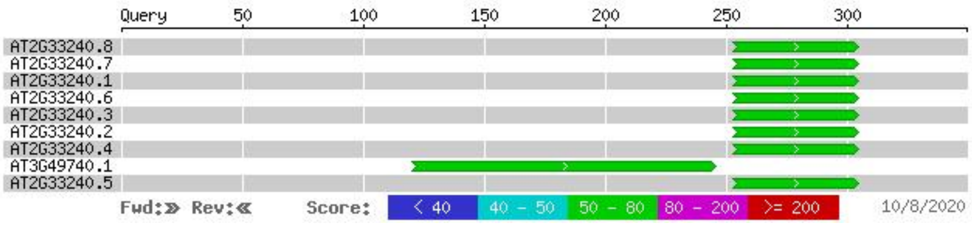

BLASTP 2.9.0+

Reference:
Stephen F. Altschul, Thomas L. Madden, Alejandro A. Schäffer, Jinghui Zhang, Zheng Zhang, Webb Miller, and David J. Lipman (1997), "Gapped BLAST and PSI-BLAST: a new generation of protein database search programs", Nucleic Acids Res. 25:3389-3402.

Reference for composition-based statistics:
Alejandro A. Schäffer, L. Aravind, Thomas L. Madden, Sergei Shavirin, John L. Spouge, Yuri I. Wolf, Eugene V. Koonin, and Stephen F. Altschul (2001), "Improving the accuracy of PSI-BLAST protein database searches with composition-based statistics and other refinements", Nucleic Acids Res. 29:2994-3005.

```
Database:  Araport11 protein sequences (protein)
           48,359 sequences; 20,855,782 total letters
```

Query= user-submitted sequence

Length=349

```
                                                                    Score     E
Sequences producing significant alignments:                         (Bits)  Value

AT2G33240.8 | Symbols:ATXID,XID | MYOSIN XI D,myosin XI D | Chr2:...  30.0    5.2
AT2G33240.7 | Symbols:ATXID,XID | MYOSIN XI D,myosin XI D | Chr2:...  30.0    5.2
AT2G33240.1 | Symbols:ATXID,XID | MYOSIN XI D,myosin XI D | Chr2:...  29.6    5.3
AT2G33240.6 | Symbols:ATXID,XID | MYOSIN XI D,myosin XI D | Chr2:...  29.6    5.4
AT2G33240.3 | Symbols:ATXID,XID | MYOSIN XI D,myosin XI D | Chr2:...  29.6    5.4
AT2G33240.2 | Symbols:ATXID,XID | MYOSIN XI D,myosin XI D | Chr2:...  29.6    5.5
AT2G33240.4 | Symbols:ATXID,XID | MYOSIN XI D,myosin XI D | Chr2:...  29.6    5.6
AT3G49740.1 | Symbols:no symbol available | no full name availabl...  28.9    9.3
```

```
AT2G33240.5 | Symbols:ATXID,XID | MYOSIN XI D,myosin XI D | Chr2:...  28.9      10.0


>AT2G33240.8 | Symbols:ATXID,XID | MYOSIN XI D,myosin XI D |  Chr2:14086942-14096914
REVERSE LENGTH=1749
Length=1749

 Score = 30.0 bits (66),  Expect = 5.2, Method: Compositional matrix adjust.
 Identities = 16/53 (30%), Positives = 26/53 (49%), Gaps = 0/53 (0%)

Query  252  MIPVLMRERGLDLQGAVDFVGRLCKGTIERFETERARLPSWGPELDAQVQTYI  304
            +I  L R++ L LQ A   +   C+G I R + +  R  +    +  Q +TYI
Sbjct  762  VITYLSRKKYLLLQSASTEIQAFCRGHIARVQFKATRREAASVRIQKQARTYI  814


>AT2G33240.7 | Symbols:ATXID,XID | MYOSIN XI D,myosin XI D |  Chr2:14086942-14096914
REVERSE LENGTH=1749
Length=1749

 Score = 30.0 bits (66),  Expect = 5.2, Method: Compositional matrix adjust.
 Identities = 16/53 (30%), Positives = 26/53 (49%), Gaps = 0/53 (0%)

Query  252  MIPVLMRERGLDLQGAVDFVGRLCKGTIERFETERARLPSWGPELDAQVQTYI  304
            +I  L R++ L LQ A   +   C+G I R + +  R  +    +  Q +TYI
Sbjct  762  VITYLSRKKYLLLQSASTEIQAFCRGHIARVQFKATRREAASVRIQKQARTYI  814


>AT2G33240.1 | Symbols:ATXID,XID | MYOSIN XI D,myosin XI D |  Chr2:14086942-14096914
REVERSE LENGTH=1770
Length=1770

 Score = 29.6 bits (65),  Expect = 5.3, Method: Compositional matrix adjust.
 Identities = 16/53 (30%), Positives = 26/53 (49%), Gaps = 0/53 (0%)

Query  252  MIPVLMRERGLDLQGAVDFVGRLCKGTIERFETERARLPSWGPELDAQVQTYI  304
            +I  L R++ L LQ A   +   C+G I R + +  R  +    +  Q +TYI
Sbjct  762  VITYLSRKKYLLLQSASTEIQAFCRGHIARVQFKATRREAASVRIQKQARTYI  814


>AT2G33240.6 | Symbols:ATXID,XID | MYOSIN XI D,myosin XI D |  Chr2:14086942-14096929
REVERSE LENGTH=1769
Length=1769

 Score = 29.6 bits (65),  Expect = 5.4, Method: Compositional matrix adjust.
 Identities = 16/53 (30%), Positives = 26/53 (49%), Gaps = 0/53 (0%)

Query  252  MIPVLMRERGLDLQGAVDFVGRLCKGTIERFETERARLPSWGPELDAQVQTYI  304
            +I  L R++ L LQ A   +   C+G I R + +  R  +    +  Q +TYI
Sbjct  767  VITYLSRKKYLLLQSASTEIQAFCRGHIARVQFKATRREAASVRIQKQARTYI  819


>AT2G33240.3 | Symbols:ATXID,XID | MYOSIN XI D,myosin XI D |  Chr2:14086942-14096914
REVERSE LENGTH=1757
Length=1757

 Score = 29.6 bits (65),  Expect = 5.4, Method: Compositional matrix adjust.
 Identities = 16/53 (30%), Positives = 26/53 (49%), Gaps = 0/53 (0%)

Query  252  MIPVLMRERGLDLQGAVDFVGRLCKGTIERFETERARLPSWGPELDAQVQTYI  304
            +I  L R++ L LQ A   +   C+G I R + +  R  +    +  Q +TYI
Sbjct  762  VITYLSRKKYLLLQSASTEIQAFCRGHIARVQFKATRREAASVRIQKQARTYI  814


>AT2G33240.2 | Symbols:ATXID,XID | MYOSIN XI D,myosin XI D |  Chr2:14086942-14096798
REVERSE LENGTH=1746
Length=1746

 Score = 29.6 bits (65),  Expect = 5.5, Method: Compositional matrix adjust.
 Identities = 16/53 (30%), Positives = 26/53 (49%), Gaps = 0/53 (0%)
```

```
Query  252  MIPVLMRERGLDLQGAVDFVGRLCKGTIERFETERARLPSWGPELDAQVQTYI  304
            +I  L R++ L LQ A   +   C+G I R + +  R  +    +  Q +TYI
Sbjct  744  VITYLSRKKYLLLQSASTEIQAFCRGHIARVQFKATRREAASVRIQKQARTYI  796


>AT2G33240.4 | Symbols:ATXID,XID | MYOSIN XI D,myosin XI D |  Chr2:14086942-14096914
REVERSE LENGTH=1764
Length=1764

 Score = 29.6 bits (65),  Expect = 5.6, Method: Compositional matrix adjust.
 Identities = 16/53 (30%), Positives = 26/53 (49%), Gaps = 0/53 (0%)

Query  252  MIPVLMRERGLDLQGAVDFVGRLCKGTIERFETERARLPSWGPELDAQVQTYI  304
            +I  L R++ L LQ A   +   C+G I R + +  R  +    +  Q +TYI
Sbjct  762  VITYLSRKKYLLLQSASTEIQAFCRGHIARVQFKATRREAASVRIQKQARTYI  814


>AT3G49740.1 | Symbols:no symbol available | no full name available |
Chr3:18447788-18450001
FORWARD LENGTH=737
Length=737

 Score = 28.9 bits (63),  Expect = 9.3, Method: Compositional matrix adjust.
 Identities = 36/135 (27%), Positives = 46/135 (34%), Gaps = 27/135 (20%)

Query  120  AFRDPVGFET--DKLGGLMSKSFFSRFRQDGGPGCTERFIHTMDLFFIAVAQQAGDRANG  177
            A RD V F    D L G         FR+       E  +   DL F++V       A G
Sbjct  253  AVRDQVTFNVVIDGLAGFKRDESLLVFRK-----MLEASLRPTDLTFVSVMGSCSCAAMG  307

Query  178  -------ITPDLESYITVRRDTSGCKPCFALIEYAAGIDLPDHVIYHPTLAAMEEATNDL  230
                   I    E Y   V   T      F            D    H    ++EE   DL
Sbjct  308  HQVHGLAIKTGYEKYTLVSNATMTMYSSF-----------EDFGAAHKVFESLEE--KDL  354

Query  231  VTWSNDIFSYNKEQV  245
            VTW+  I SYN+ ++
Sbjct  355  VTWNTMISSYNQAKL  369


>AT2G33240.5 | Symbols:ATXID,XID | MYOSIN XI D,myosin XI D |  Chr2:14086942-14093527
REVERSE LENGTH=1243
Length=1243

 Score = 28.9 bits (63),  Expect = 10.0, Method: Composition-based stats.
 Identities = 16/53 (30%), Positives = 26/53 (49%), Gaps = 0/53 (0%)

Query  252  MIPVLMRERGLDLQGAVDFVGRLCKGTIERFETERARLPSWGPELDAQVQTYI  304
            +I  L R++ L LQ A   +   C+G I R + +  R  +    +  Q +TYI
Sbjct  235  VITYLSRKKYLLLQSASTEIQAFCRGHIARVQFKATRREAASVRIQKQARTYI  287



Lambda      K        H        a         alpha
   0.323    0.137    0.437    0.792     4.96

Gapped
Lambda      K        H        a         alpha    sigma
   0.267   0.0410    0.140     1.90     42.6     43.6

Effective search space used: 3877479270


  Database:  Araport11 protein sequences (protein)
    Posted date:  May 5, 2018  8:08 AM
  Number of letters in database: 20,855,782
  Number of sequences in database:  48,359
```

```
Matrix: BLOSUM62
Gap Penalties: Existence: 11, Extension: 1
Neighboring words threshold: 11
Window for multiple hits: 40
```

printer-friendly version

General comments or questions: curator@arabidopsis.org
Seed or DNA stock questions (donations, availability, orders, etc):abrc@osu.edu
