# Supplementary material for: Fungal genomes: suffering with functional annotation errors
Source: IMA Fungus. 2021 Nov 1;12:32. doi: 10.1186/s43008-021-00083-x (PMC8559351; doi:10.1186/s43008-021-00083-x)
Supplement: Supplementary file 10 — Additional file 10: Fig. 3. BLASTP analysis of fungal proteins annotated with the term “sesquiterpene cyclase” (accession number XP_020126298.1). BLASTP results identified a radiation sensitive 23C DNA repair protein (RAD23-3) rather than a sesquiterpene cyclase. [file 43008_2021_83_MOESM10_ESM.pdf]

blastp query on Araport11 protein sequences (protein) sequences

Query performed by the The Arabidopsis Information Resource (TAIR); for full BLAST options and parameters, refer to the NCBI BLAST Documentation

Your comments and suggestions are requested: Send a Message to TAIR

Summary of BLAST Results Help

All hits shown.

| Symbols:no symbol available | no full name available | Chr5:373014-374651 REVERSE LENGTH=351

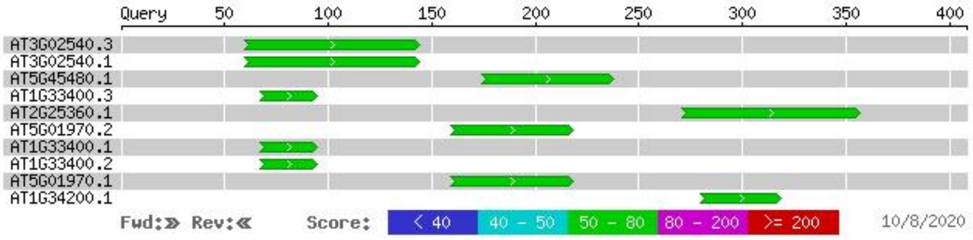

BLASTP 2.9.0+

Reference:
Stephen F. Altschul, Thomas L. Madden, Alejandro A. Schäffer, Jinghui Zhang, Zheng Zhang, Webb Miller, and David J. Lipman (1997), "Gapped BLAST and PSI-BLAST: a new generation of protein database search programs", Nucleic Acids Res. 25:3389-3402.

Reference for composition-based statistics:
Alejandro A. Schäffer, L. Aravind, Thomas L. Madden, Sergei Shavirin, John L. Spouge, Yuri I. Wolf, Eugene V. Koonin, and Stephen F. Altschul (2001), "Improving the accuracy of PSI-BLAST protein database searches with composition-based statistics and other refinements", Nucleic Acids Res. 29:2994-3005.

Database: Araport11 protein sequences (protein)
48,359 sequences; 20,855,782 total letters

Query= user-submitted sequence

Length=408

Sequences producing significant alignments:	Score (Bits)	E Value
AT3G02540.3 | Symbols:RAD23-3,RAD23C | RADIATION SENSITIVE23C,PUT...	30.8	2.6
AT3G02540.1 | Symbols:RAD23-3,RAD23C | RADIATION SENSITIVE23C,PUT...	30.8	2.7
AT5G45480.1 | Symbols:no symbol available | no full name availabl...	30.4	4.1
AT1G33400.3 | Symbols:TPR9 | tetratricopeptide repeat 9 | Chr1:12...	30.0	5.0
AT2G25360.1 | Symbols:no symbol available | no full name availabl...	29.6	5.3
AT5G01970.2 | Symbols:no symbol available | no full name availabl...	29.6	5.5
AT1G33400.1 | Symbols:TPR9 | tetratricopeptide repeat 9 | Chr1:12...	30.0	5.8

```
AT1G33400.2 | Symbols:TPR9 | tetratricopeptide repeat 9 | Chr1:12...  30.0       5.9
AT5G01970.1 | Symbols:no symbol available | no full name availabl...  29.6       6.5
AT1G34200.1 | Symbols:no symbol available | no full name availabl...  28.9       9.5


>AT3G02540.3 | Symbols:RAD23-3,RAD23C | RADIATION SENSITIVE23C,PUTATIVE DNA
REPAIR PROTEIN RAD23-3 |  Chr3:533095-536151 REVERSE LENGTH=418

Length=418

 Score = 30.8 bits (68),  Expect = 2.6, Method: Compositional matrix adjust.
 Identities = 25/93 (27%), Positives = 41/93 (44%), Gaps = 16/93 (17%)

Query  59   PATASSNSKAANWIDIFP-------ADAGIASRTALRVFRQSKYWRTVLSAGSDALQHFL  111
            PA A ++   AN +D+FP        + G  +   LR  +Q +  R ++ A    LQ  L
Sbjct  257  PAAAPASGPNANPLDLFPQGLPNVGGNPGAGTLDFLRNSQQFQALRAMVQANPQVLQPML  316

Query  112  QDDSFKQAVRSGKTFSKISAKLLQNIEDGFLRF  144
            Q+          GK    +  +L+Q+ +  FLR
Sbjct  317  QE--------LGKQNPNL-MRLIQDHQADFLRL  340


>AT3G02540.1 | Symbols:RAD23-3,RAD23C | RADIATION SENSITIVE23C,PUTATIVE DNA
REPAIR PROTEIN RAD23-3 |  Chr3:533095-536151 REVERSE LENGTH=419

Length=419

 Score = 30.8 bits (68),  Expect = 2.7, Method: Compositional matrix adjust.
 Identities = 25/93 (27%), Positives = 41/93 (44%), Gaps = 16/93 (17%)

Query  59   PATASSNSKAANWIDIFP-------ADAGIASRTALRVFRQSKYWRTVLSAGSDALQHFL  111
            PA A ++   AN +D+FP        + G  +   LR  +Q +  R ++ A    LQ  L
Sbjct  258  PAAAPASGPNANPLDLFPQGLPNVGGNPGAGTLDFLRNSQQFQALRAMVQANPQVLQPML  317

Query  112  QDDSFKQAVRSGKTFSKISAKLLQNIEDGFLRF  144
            Q+          GK    +  +L+Q+ +  FLR
Sbjct  318  QE--------LGKQNPNL-MRLIQDHQADFLRL  341


>AT5G45480.1 | Symbols:no symbol available | no full name available |
Chr5:18426296-18428929
REVERSE LENGTH=877
Length=877

 Score = 30.4 bits (67),  Expect = 4.1, Method: Compositional matrix adjust.
 Identities = 16/64 (25%), Positives = 30/64 (47%), Gaps = 0/64 (0%)

Query  174  WEMEDGEEVTKLRDDFVSRLQTSDKAGESPAETSTKTPLQAMIDQSIAELRQFSSTGGQE  233
            W +E+   V KL+ +       + ++ E P  ++ KTP    I   I  +R+  +    E
Sbjct  382  WILENILGVRKLKVEEYDECYKNTQSHEVPNTSTKKTPFLKRILNRILRVRELKTEKSHE  441

Query  234  VIDE  237
            V+D+
Sbjct  442  VLDK  445


>AT1G33400.3 | Symbols:TPR9 | tetratricopeptide repeat 9 |  Chr1:12104891-12108719
REVERSE LENGTH=632
Length=632

 Score = 30.0 bits (66),  Expect = 5.0, Method: Compositional matrix adjust.
 Identities = 10/28 (36%), Positives = 17/28 (61%), Gaps = 0/28 (0%)

Query  67   KAANWIDIFPADAGIASRTALRVFRQSK  94
            + ANW  + P+DA +A R  +++  Q K
Sbjct  178  RGANWPAVLPSDAVLAGRIIMKLINQGK  205


>AT2G25360.1 | Symbols:no symbol available | no full name available |
```

```
Chr2:10804249-10805761
FORWARD LENGTH=373
Length=373

 Score = 29.6 bits (65),  Expect = 5.3, Method: Compositional matrix adjust.
 Identities = 22/88 (25%), Positives = 35/88 (40%), Gaps = 7/88 (8%)

Query  270  CILACTKFSIDSTVDIHSP-RMAKIIELMGNHMIFANDLGSFAKEKRAFSAGKIQHLINS  328
            C+  C  F ID  V  HS    A+  +L  + ++    L S AK+K+       +H+I
Sbjct  203  CVKCCGLFCIDCKVPSHSDLSCAEYKKLHHDPLVDELKLKSLAKDKKWRQCKMCRHMIEL  262

Query  329  VHVVKQLLGLPSDEAAKGVVYGLQLQVE  356
             H    +           G  +  Q +VE
Sbjct  263  SHACNHMT------CRCGYQFCYQCEVE  284


>AT5G01970.2 | Symbols:no symbol available | no full name available |
Chr5:373014-374028
REVERSE LENGTH=254
Length=254

 Score = 29.6 bits (65),  Expect = 5.5, Method: Compositional matrix adjust.
 Identities = 23/61 (38%), Positives = 32/61 (52%), Gaps = 7/61 (11%)

Query  159  LLAIVMVYIFVFDDLWEMEDG-EEVTKLRDDFVSRLQTSDKAGESPAETSTKTPLQAMID  217
            L  IV  +     D+  +++G EEVT++    FVS L TS      SPA+ S    P Q MI
Sbjct  182  LREIVEYHQLTMQDVVYIDEGSEEVTQV-SPFVSTLMTS-----SPADRSQSPPSQEMIK  235

Query  218  Q  218
            +
Sbjct  236  E  236


>AT1G33400.1 | Symbols:TPR9 | tetratricopeptide repeat 9 |  Chr1:12104891-12109488
REVERSE LENGTH=798
Length=798

 Score = 30.0 bits (66),  Expect = 5.8, Method: Compositional matrix adjust.
 Identities = 10/28 (36%), Positives = 17/28 (61%), Gaps = 0/28 (0%)

Query  67   KAANWIDIFPADAGIASRTALRVFRQSK  94
            + ANW  + P+DA +A R  +++  Q K
Sbjct  344  RGANWPAVLPSDAVLAGRIIMKLINQGK  371


>AT1G33400.2 | Symbols:TPR9 | tetratricopeptide repeat 9 |  Chr1:12104891-12109424
REVERSE LENGTH=810
Length=810

 Score = 30.0 bits (66),  Expect = 5.9, Method: Compositional matrix adjust.
 Identities = 10/28 (36%), Positives = 17/28 (61%), Gaps = 0/28 (0%)

Query  67   KAANWIDIFPADAGIASRTALRVFRQSK  94
            + ANW  + P+DA +A R  +++  Q K
Sbjct  356  RGANWPAVLPSDAVLAGRIIMKLINQGK  383


>AT5G01970.1 | Symbols:no symbol available | no full name available |
Chr5:373014-374651
REVERSE LENGTH=351
Length=351

 Score = 29.6 bits (65),  Expect = 6.5, Method: Compositional matrix adjust.
 Identities = 23/61 (38%), Positives = 32/61 (52%), Gaps = 7/61 (11%)

Query  159  LLAIVMVYIFVFDDLWEMEDG-EEVTKLRDDFVSRLQTSDKAGESPAETSTKTPLQAMID  217
            L  IV  +     D+  +++G EEVT++    FVS L TS      SPA+ S    P Q MI
Sbjct  279  LREIVEYHQLTMQDVVYIDEGSEEVTQV-SPFVSTLMTS-----SPADRSQSPPSQEMIK  332
```

```
Query  218  Q  218
            +
Sbjct  333  E  333


>AT1G34200.1 | Symbols:no symbol available | no full name available |
Chr1:12455793-12456935
FORWARD LENGTH=352
Length=352

 Score = 28.9 bits (63),  Expect = 9.5, Method: Compositional matrix adjust.
 Identities = 17/40 (43%), Positives = 21/40 (53%), Gaps = 7/40 (18%)

Query  279  IDSTVDIHSPRMAKIIELMGNHMIFANDLGSFAKEKRAFS  318
            +D T  +HSPR  KI E       F NDL SF + K  +S
Sbjct  127  MDGTQWMHSPRTDKIKE-------FVNDLESFGQIKSVYS  159



Lambda      K        H        a         alpha
   0.321    0.134    0.388    0.792     4.96

Gapped
Lambda      K        H        a         alpha    sigma
   0.267   0.0410    0.140     1.90     42.6     43.6

Effective search space used: 4780760361


  Database:  Araport11 protein sequences (protein)
    Posted date:  May 5, 2018  8:08 AM
  Number of letters in database: 20,855,782
  Number of sequences in database:  48,359



Matrix: BLOSUM62
Gap Penalties: Existence: 11, Extension: 1
Neighboring words threshold: 11
Window for multiple hits: 40
```

printer-friendly version

General comments or questions: curator@arabidopsis.org
Seed or DNA stock questions (donations, availability, orders, etc):abrc@osu.edu
