# Supplementary material for: Fungal genomes: suffering with functional annotation errors
Source: IMA Fungus. 2021 Nov 1;12:32. doi: 10.1186/s43008-021-00083-x (PMC8559351; doi:10.1186/s43008-021-00083-x)
Supplement: Supplementary file 11 — Additional file 11: Fig. 4. BLASTP analysis of a fungal protein annotated as a leghemoglobin (accession number KXX77420.1) protein against proteins in the NCBI database. Results only identified a hit to itself (KXX77420.1) and no other protein was found to be a leghemoglobin; indicating the erroneous annotation of fungal proteins. [file 43008_2021_83_MOESM11_ESM.pdf]

COVID-19 is an emerging, rapidly evolving situation.
Get the latest public health information from CDC: https://www.coronavirus.gov .
Get the latest research from NIH: https://www.nih.gov/coronavirus.
Find NCBI SARS-CoV-2 literature, sequence, and clinical content: https://www.ncbi.nlm.nih.gov/sars-cov-2/.

BLAST ® » blastp suite » results for RID-RXZ03FMJ014

**Job Title**	Protein Sequence ...
**RID**	RXZ03FMJ014 Search expires on 10-09 19:46 pm
**Program**	Quick BLASTP
**Database**	nr
**Query ID**	lcl|Query_66737
**Description**	None ...
**Molecule type**	amino acid
**Query Length**	465

Descriptions

Description	Max Score	Total Score	Query Cover	E value	Per. Ident	Accession
Leghemoglobin reductase [Madurella mycetomatis]	932	932	100%	0.0	100.00%	KXX77420.1
hypothetical protein CHGG_00138 [Chaetomium globosum CBS 148.51]	721	721	100%	0.0	74.31%	XP_001219359.1
putative dihydrolipoyl dehydrogenase protein [Phaeoacremonium minimum UCRPA7]	581	581	99%	0.0	59.06%	XP_007915022.1
FAD/NAD(P)-binding domain-containing protein [Coniochaeta ligniaria NRRL 30616]	574	574	98%	0.0	60.34%	OIW30451.1
hypothetical protein GE09DRAFT_1064680 [Coniochaeta sp. 2T2.1]	565	565	98%	0.0	59.70%	KAB5527974.1
hypothetical protein GE09DRAFT_15414 [Coniochaeta sp. 2T2.1]	555	555	98%	0.0	60.34%	KAB5585116.1
hypothetical protein EG329_003274 [Helotiales sp. DMI_Dod_QoI]	508	508	99%	1e-174	56.45%	KAE8442493.1
hypothetical protein B0A52_07332 [Exophiala mesophila]	496	496	99%	3e-170	54.12%	RVX68329.1
FAD-dependent pyridine nucleotide-disulfide oxidoreductase [Trematosphaeria pertusa]	488	488	99%	8e-167	52.33%	XP_033688688.1
Dihydrolipoyl dehydrogenase [Hyaloscypha variabilis F]	481	481	99%	6e-164	53.96%	PMD29251.1
hypothetical protein GGTG_11046 [Gaeumannomyces tritici R3-111a-1]	479	479	99%	3e-163	54.18%	XP_009227190.1
FAD-dependent pyridine nucleotide-disulfide oxidoreductase [Clohesyomyces aquaticus]	478	478	99%	5e-163	53.62%	ORY16189.1
FAD/NAD(P)-binding domain-containing protein [Chalara longipes BDJ]	476	476	99%	3e-162	52.78%	KAE9375044.1
FAD-dependent oxidoreductase [Planctomyces sp. SH-PL62]	476	476	99%	3e-162	53.00%	WP_068422479.1
Dihydrolipoyl dehydrogenase [Hyaloscypha bicolor E]	476	476	98%	6e-162	53.00%	XP_024741558.1
FAD-dependent oxidoreductase [Paludisphaera sp. JC665]	473	473	99%	3e-161	54.49%	WP_165070541.1
FAD-dependent pyridine nucleotide-disulfide oxidoreductase [Lophiostoma macrostomum CBS 122681]	471	471	98%	2e-160	51.72%	KAF2658962.1
FAD-dependent pyridine nucleotide-disulfide oxidoreductase [Cenococcum geophilum 1.58]	470	470	99%	1e-159	51.71%	OCK89743.1
FAD-dependent pyridine nucleotide-disulfide oxidoreductase [Granulicella tundricola MP5ACTX9]	470	470	99%	2e-159	52.23%	ADW69689.1
FAD-dependent oxidoreductase [Granulicella tundricola]	469	469	99%	2e-159	52.23%	WP_041597111.1
FAD-dependent oxidoreductase [Planctomycetes bacterium ElP]	468	468	99%	5e-159	53.43%	WP_145272511.1

Description	Max Score	Total Score	Query Cover	E value	Per. Ident	Accession
FAD/NAD(P)-binding protein [Glarea lozoyensis ATCC 20868]	468	468	99%	7e-159	51.59%	XP_008075901.1
Dihydrolipoyl dehydrogenase [Stagonospora sp. SRC1lsM3a]	468	468	99%	8e-159	52.88%	OAK94831.1
FAD-dependent oxidoreductase [Gemmata massiliana]	466	466	99%	5e-158	52.03%	WP_162672821.1
FAD-dependent oxidoreductase [Gemmata sp. SH-PL17]	464	464	99%	1e-157	52.03%	WP_082838148.1
Dihydrolipoyl dehydrogenase [Pyrenochaeta sp. DS3sAY3a]	464	464	99%	3e-157	51.58%	OAL51325.1
Dihydrolipoyl dehydrogenase [Ophiobolus disseminans]	461	461	98%	2e-156	52.46%	KAF2824058.1
FAD-dependent oxidoreductase [Terrimicrobium sacchariphilum]	461	461	99%	3e-156	52.03%	WP_075080549.1
FAD-dependent oxidoreductase [Silvibacterium bohemicum]	461	461	99%	3e-156	51.60%	WP_050061725.1
pyruvate/2-oxoglutarate dehydrogenase complex [Terrimicrobium sacchariphilum]	460	460	99%	8e-156	51.81%	GAT31921.1
Dihydrolipoyl dehydrogenase [Pezoloma ericae]	457	457	99%	2e-154	51.28%	PMD16242.1
FAD-dependent oxidoreductase [Lacipirellula parvula]	456	456	98%	3e-154	52.35%	WP_152100060.1
FAD-dependent oxidoreductase [Zavarzinella formosa]	456	456	99%	3e-154	53.10%	WP_020474773.1
FAD-dependent oxidoreductase [Edaphobacter aggregans]	453	453	99%	3e-153	49.57%	WP_125484885.1
FAD-dependent oxidoreductase [Singulisphaera sp. GP187]	452	452	99%	4e-153	51.72%	WP_074308700.1
mercuric reductase [Verrucomicrobia bacterium]	453	453	99%	6e-153	49.25%	PYK02771.1
FAD-dependent pyridine nucleotide-disulfide oxidoreductase [Rhizodiscina lignyota]	452	452	99%	2e-152	51.66%	KAF2093634.1
FAD-dependent oxidoreductase [Singulisphaera acidiphila]	448	448	99%	2e-151	51.50%	WP_015247128.1
Pyruvate/2-oxoglutarate dehydrogenase complex, dihydrolipoamide dehydrogenase (E3) component [Singulisphaera sp. GP187]	445	445	99%	1e-149	51.39%	SIO66840.1
FAD-dependent oxidoreductase [Singulisphaera sp. GP187]	444	444	99%	1e-149	51.39%	WP_074317373.1
putative pyridine nucleotide-disulfide oxidoreductase RclA [Paludisphaera borealis]	444	444	99%	2e-149	50.54%	APW61714.1
FAD-dependent oxidoreductase [Granulicella mallensis]	442	442	98%	1e-148	49.36%	WP_184254370.1
FAD-dependent oxidoreductase [Paludisphaera borealis]	441	441	99%	2e-148	50.32%	WP_076350975.1
pyruvate/2-oxoglutarate dehydrogenase complex dihydrolipoamide dehydrogenase (E3) component [Granulicella mallensis]	442	442	98%	2e-148	49.36%	MBB5063034.1
FAD-dependent oxidoreductase [Paraburkholderia sp. RP-4-7]	442	442	98%	3e-148	48.82%	WP_169491108.1
FAD-dependent oxidoreductase [Granulicella sp. WH15]	440	440	98%	5e-148	51.30%	WP_162539072.1
FAD-dependent oxidoreductase [Granulicella sp. S190]	438	438	99%	2e-147	48.29%	WP_158943510.1
FAD-dependent oxidoreductase [Acidobacteriaceae bacterium KBS 146]	437	437	99%	5e-147	49.25%	WP_026388560.1
FAD-dependent oxidoreductase [Frigoriglobus tundricola]	437	437	99%	6e-147	49.25%	WP_171470804.1
FAD-dependent oxidoreductase [Edaphobacter sp. 12200R-103]	437	437	99%	7e-147	47.76%	WP_162402250.1
FAD/NAD(P)-binding domain-containing protein [Hyaloscypha variabilis F]	433	433	82%	3e-146	56.63%	PMD31504.1
FAD-dependent oxidoreductase [Granulicella sp. GAS466]	434	434	99%	2e-145	49.25%	WP_123489827.1
FAD-dependent oxidoreductase [Granulicella sp. L60]	434	434	98%	2e-145	48.81%	WP_158790451.1
FAD-dependent oxidoreductase [Gemmata obscuriglobus]	432	432	98%	6e-145	51.50%	WP_010053189.1
FAD-dependent oxidoreductase [Singulisphaera sp. GP187]	429	429	99%	1e-143	49.68%	WP_074316341.1

Description	Max Score	Total Score	Query Cover	E value	Per. Ident	Accession
uncharacterized protein M409DRAFT_64983 [Zasmidium cellare ATCC 36951]	426	426	99%	6e-143	48.63%	XP_033670150.1
Pyruvate/2-oxoglutarate dehydrogenase complex, dihydrolipoamide dehydrogenase (E3) component [Terriglobus roseus]	426	426	98%	2e-142	48.60%	SDF98400.1
FAD-dependent oxidoreductase [Acidobacterium sp. 4Y35]	426	426	98%	2e-142	48.93%	WP_186745562.1
FAD-dependent oxidoreductase [Terriglobus roseus]	426	426	98%	3e-142	48.60%	WP_156785218.1
FAD-dependent oxidoreductase [Gemmata massiliana]	422	422	98%	5e-141	50.86%	WP_162670453.1
FAD-dependent oxidoreductase [Planctomycetes bacterium ElP]	419	419	99%	5e-140	49.36%	WP_145272489.1
mercuric reductase [Methylobacterium sp. 174MFSha1.1]	416	416	98%	8e-139	47.97%	WP_093569215.1
FAD-dependent oxidoreductase [Acetobacter nitrogenifigens]	416	416	100%	2e-138	48.83%	WP_026397664.1
FAD-dependent oxidoreductase [Capsulimonas corticalis]	415	415	99%	3e-138	47.97%	WP_119321024.1
FAD-dependent oxidoreductase [Acidisarcina polymorpha]	412	412	98%	6e-137	46.67%	WP_114205464.1
mercuric reductase [Methylobacterium sp. 6HR-1]	411	411	98%	9e-137	47.97%	WP_135416742.1
Pyruvate/2-oxoglutarate dehydrogenase complex, dihydrolipoamide dehydrogenase (E3) component [Bryocella elongata]	411	411	98%	1e-136	49.46%	SEG03116.1
FAD-dependent oxidoreductase [Bryocella elongata]	410	410	98%	2e-136	49.46%	WP_103932789.1
uncharacterized protein SETTUDRAFT_159632 [Exserohilum turcica Et28A]	411	411	96%	2e-136	47.08%	XP_008023056.1
mercuric reductase [Mesorhizobium ciceri]	410	410	100%	3e-136	48.84%	WP_027035822.1
mercuric reductase [Methylobacterium sp. 17Sr1-43]	408	408	98%	1e-135	47.73%	WP_109952220.1
mercuric reductase [Methylobacterium variabile]	408	408	98%	2e-135	48.17%	WP_048443666.1
mercuric reductase [Rhodanobacter fulvus]	407	407	99%	6e-135	47.33%	WP_007081217.1
FAD-dependent oxidoreductase [Granulicella sibirica]	406	406	98%	7e-135	47.95%	WP_128912808.1
mercuric reductase [Pseudaminobacter manganicus]	406	406	99%	7e-135	46.81%	WP_080921142.1
mercuric reductase [Acidobacteria bacterium]	406	406	99%	1e-134	47.46%	PYU19249.1
mercuric reductase [Acidobacteria bacterium]	405	405	99%	1e-134	48.40%	PYQ03069.1
FAD-dependent oxidoreductase [Aquisphaera sp. JC669]	405	405	97%	1e-134	49.02%	WP_165224774.1
FAD-dependent oxidoreductase [Granulicella sibirica]	405	405	98%	2e-134	46.02%	WP_128914270.1
FAD-dependent oxidoreductase [Fimbriiglobus ruber]	405	405	97%	3e-134	46.87%	WP_088259766.1
mercuric reductase [Rhodanobacter sp. OR444]	404	404	98%	8e-134	48.28%	WP_027491238.1
mercuric reductase [Methylobacterium tarhaniae]	403	403	98%	1e-133	48.16%	WP_048454291.1
mercuric reductase [Rhodanobacter denitrificans]	403	403	99%	2e-133	47.76%	WP_114340465.1
FAD-dependent oxidoreductase [Opitutus sp. ER46]	402	402	98%	6e-133	47.42%	WP_107835729.1
mercuric reductase [Mesorhizobium sp. B2-3-3]	400	400	100%	2e-132	47.35%	TPN23623.1
FAD-dependent oxidoreductase [Acidobacteria bacterium Pan2503]	398	398	90%	2e-132	49.88%	MBA0087722.1
FAD-dependent oxidoreductase [Mesorhizobium sp. B2-3-5]	400	400	100%	4e-132	47.13%	WP_140747153.1
FAD-dependent oxidoreductase [Mesorhizobium sp. B2-4-15]	399	399	100%	8e-132	47.35%	WP_141840360.1
mercuric reductase [Acidobacteria bacterium]	399	399	98%	1e-131	45.40%	PYX73050.1
mercuric reductase [Rhodanobacter thiooxydans]	398	398	98%	2e-131	47.63%	WP_008438212.1
mercuric reductase [Duganella sp. DN04]	396	396	99%	6e-131	45.74%	WP_135202832.1
mercuric reductase [Pseudomonas acidophila]	396	396	98%	9e-131	46.35%	PCE23994.1
FAD-dependent oxidoreductase [Paraburkholderia caledonica]	394	394	99%	4e-130	45.96%	WP_087753710.1
FAD-dependent oxidoreductase [Mesorhizobium sp. B4-1-4]	394	394	99%	9e-130	46.17%	WP_140766259.1
mercuric reductase [Pirellula sp.]	392	392	97%	2e-129	46.85%	MBA4105228.1
mercuric reductase [Burkholderia sp. WSM2230]	392	392	99%	2e-129	45.74%	WP_025598434.1
mercuric reductase [Paraburkholderia caledonica]	392	392	99%	3e-129	45.74%	WP_020067768.1
mercuric reductase [Mesorhizobium sp. B2-4-12]	391	391	99%	7e-129	45.96%	WP_140610964.1

Description	Max Score	Total Score	Query Cover	E value	Per. Ident	Accession
FAD-dependent oxidoreductase [Methylobacterium crusticola]	391	391	98%	8e-129	49.57%	WP_128565516.1
FAD-dependent oxidoreductase [Mesorhizobium sp. B2-4-17]	390	390	99%	1e-128	45.96%	WP_140776343.1

Graphic Summary

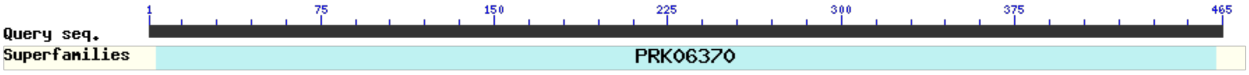

Distribution of the top 100 Blast Hits on 100 subject sequences

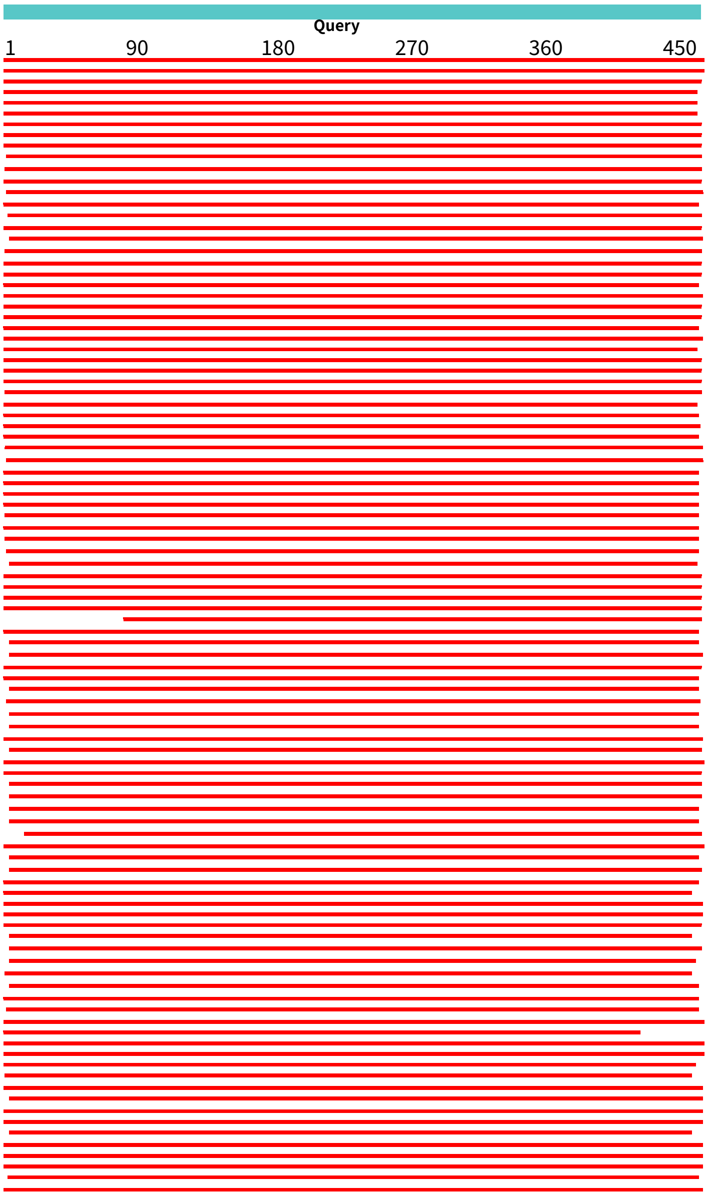

Alignments

Alignment view Pairwise CDS feature Restore defaults

Leghemoglobin reductase [Madurella mycetomatis]

Sequence ID: **KXX77420.1** Length: 465 Number of Matches: 1

Range 1: 1 to 465

Score	Expect	Method	Identities	Positives	Gaps	Frame
932 bits(2410)	0.0()	Compositional matrix adjust.	465/465(100%)	465/465(100%)	0/465(0%)	

```
Query  1     MASPEQFDFVALGAGEPAKLLAWDLSSNHGMKCAVIEHGPIAGSCPTVACMPSKTIIHSA  60
             MASPEQFDFVALGAGEPAKLLAWDLSSNHGMKCAVIEHGPIAGSCPTVACMPSKTIIHSA
Sbjct  1     MASPEQFDFVALGAGEPAKLLAWDLSSNHGMKCAVIEHGPIAGSCPTVACMPSKTIIHSA  60

Query  61    HVAHLARQAPAFGIGAARDDVKADMTAVVARKKAVVDGMADLFLGIFADTKVELIRGHGE  120
             HVAHLARQAPAFGIGAARDDVKADMTAVVARKKAVVDGMADLFLGIFADTKVELIRGHGE
Sbjct  61    HVAHLARQAPAFGIGAARDDVKADMTAVVARKKAVVDGMADLFLGIFADTKVELIRGHGE  120

Query  121   LAGPKTVRVNGRLLTGNTVLINTGSKAFVDTKIPGLVDAKPLTHVELLNLTTLPSHLIIL  180
             LAGPKTVRVNGRLLTGNTVLINTGSKAFVDTKIPGLVDAKPLTHVELLNLTTLPSHLIIL
Sbjct  121   LAGPKTVRVNGRLLTGNTVLINTGSKAFVDTKIPGLVDAKPLTHVELLNLTTLPSHLIIL  180

Query  181   GGGYVGLEFAQAFVRFGAQVTVVERNSRVLKNEDDDVVASLTAILAREGVQFLTSTSVAR  240
             GGGYVGLEFAQAFVRFGAQVTVVERNSRVLKNEDDDVVASLTAILAREGVQFLTSTSVAR
Sbjct  181   GGGYVGLEFAQAFVRFGAQVTVVERNSRVLKNEDDDVVASLTAILAREGVQFLTSTSVAR  240

Query  241   VSGTSGTEVVLTLSGPGPAEIRGSHLLVATGRTPNTSGIGLVEAGIKLSNTGHVAVDEQL  300
             VSGTSGTEVVLTLSGPGPAEIRGSHLLVATGRTPNTSGIGLVEAGIKLSNTGHVAVDEQL
Sbjct  241   VSGTSGTEVVLTLSGPGPAEIRGSHLLVATGRTPNTSGIGLVEAGIKLSNTGHVAVDEQL  300

Query  301   GSSVPGVFAAGDCAGSPLFTHMGWDDYRIILASLTGSPREGGKTTRQVPSTLFTSPELAH  360
             GSSVPGVFAAGDCAGSPLFTHMGWDDYRIILASLTGSPREGGKTTRQVPSTLFTSPELAH
Sbjct  301   GSSVPGVFAAGDCAGSPLFTHMGWDDYRIILASLTGSPREGGKTTRQVPSTLFTSPELAH  360

Query  361   VGLREEEAKAKGIPYRLTKAPMAAFLRTRALGETDGFAKALVEADGDRVLGFTALGPSAG  420
             VGLREEEAKAKGIPYRLTKAPMAAFLRTRALGETDGFAKALVEADGDRVLGFTALGPSAG
Sbjct  361   VGLREEEAKAKGIPYRLTKAPMAAFLRTRALGETDGFAKALVEADGDRVLGFTALGPSAG  420

Query  421   ELLPAVQLVMKLGLSYKELVDLVLVHPTMCEGLVDLFRSVPPRAK  465
             ELLPAVQLVMKLGLSYKELVDLVLVHPTMCEGLVDLFRSVPPRAK
Sbjct  421   ELLPAVQLVMKLGLSYKELVDLVLVHPTMCEGLVDLFRSVPPRAK  465
```

hypothetical protein CHGG_00138 [Chaetomium globosum CBS 148.51]

Sequence ID: **XP_001219359.1** Length: 471 Number of Matches: 1

Range 1: 1 to 471

Score	Expect	Method	Identities	Positives	Gaps	Frame
721 bits(1860)	0.0()	Compositional matrix adjust.	350/471(74%)	396/471(84%)	6/471(1%)	

```
Query  1     MASPEQFDFVALGAGEPAKLLAWDLSSNHGMKCAVIEHGPIAGSCPTVACMPSKTIIHSA  60
             MA+PEQ+DF+ALG GEPAKLLAWDLSS +G KCAVIEHGPI+G+CPTVACMP+KT++HSA
Sbjct  1     MATPEQYDFIALGGGEPAKLLAWDLSSKYGKKCAVIEHGPISGACPTVACMPTKTLLHSA  60

Query  61    HVAHLARQAPAFGIGAARDDVKADMTAVVARKKAVVDGMADLFLGIFADTKVELIRGHGE  120
              +AHLARQA A   GAA +   ADM  V ARK+ VVDGMADLFLGIFA+TK ELIRGHGE
Sbjct  61    QLAHLARQAQASTPGAAGNGFNADMAKVFARKQEVVDGMADLFLGIFAETKAELIRGHGE  120

Query  121   LAGPKTVRVNGRLLTGNTVLINTGSKAFVDTKIPGLVDAKPLTHVELLNLTTLPSHLIIL  180
                PKT+  NGRLLT  TVLINTGSKAFVDT IPGL DA PLTHVELL++ TLPSHLIIL
Sbjct  121   FVDPKTISCNGRLLTAETVLINTGSKAFVDTSIPGLADANPLTHVELLDIKTLPSHLIIL  180

Query  181   GGGYVGLEFAQAFVRFGAQVTVVERNSRVLKNEDDDVVASLTAILAREGVQFLTSTSVAR  240
             GGGYVG+EFAQA+ RFG++VTV+ERN+++L  ED DVVA LT +LAREG+ FLTSTSV
Sbjct  181   GGGYVGIEFAQAYARFGSRVTVIERNAQILAKEDSDVVAELTRLLAREGIDFLTSTSVTH  240

Query  241   VSGTSGTEVVLTLSGP------GPAEIRGSHLLVATGRTPNTSGIGLVEAGIKLSNTGHV  294
             VSGTSG+EV LTLS P       P  IRG+HLLVA GRTP T+ +GL  AGIKL+ TGH+
Sbjct  241   VSGTSGSEVTLTLSHPTPGAGAAPTSIRGTHLLVAAGRTPTTANLGLAAAGIKLTPTGHI  300

Query  295   AVDEQLGSSVPGVFAAGDCAGSPLFTHMGWDDYRIILASLTGSPREGGKTTRQVPSTLFT  354
             AVD QL +SVPGVFAAGDCAGSP FTHMGWDDYR++L  +TG+PRE G   RQVPS LFT
Sbjct  301   AVDAQLRTSVPGVFAAGDCAGSPYFTHMGWDDYRVLLGVVTGAPREAGTMGRQVPSVLFT  360

Query  355   SPELAHVGLREEEAKAKGIPYRLTKAPMAAFLRTRALGETDGFAKALVEADGDRVLGFTA  414
             +PELAHVGLREEEAK KG+ YR+ +APM AFLR RALGET+GFAKALVE +G+RVLGFTA
Sbjct  361   TPELAHVGLREEEAKKKGVGYRVVRAPMGAFLRARALGETEGFAKALVEEEGERVLGFTA  420

Query  415   LGPSAGELLPAVQLVMKLGLSYKELVDLVLVHPTMCEGLVDLFRSVPPRAK  465
             LGP AGELLP VQLVMKLGLSYKELVDL +VHPTM EGLVDLFRSVPP +K
Sbjct  421   LGPGAGELLPVVQLVMKLGLSYKELVDLTIVHPTMAEGLVDLFRSVPPSSK  471
```

putative dihydrolipoyl dehydrogenase protein [Phaeoacremonium minimum UCRPA7]

Sequence ID: **XP_007915022.1** Length: 471 Number of Matches: 1

Range 1: 1 to 469

Score	Expect	Method	Identities	Positives	Gaps	Frame
581 bits(1498)	0.0()	Compositional matrix adjust.	277/469(59%)	360/469(76%)	6/469(1%)	

```
Query  1     MASPEQFDFVALGAGEPAKLLAWDLSSNHGMKCAVIEHGPIAGSCPTVACMPSKTIIHSA  60
             M++PE FD++++G+GE  K +AW+LS+N G +CAVIE   I GSCP +AC+PSK  +HSA
Sbjct  1     MSAPEHFDYISIGSGEAGKYIAWNLSANQGKRCAVIERKWIGGSCPNIACLPSKNFLHSA  60

Query  61    HVAHLARQAPAFGIGA---ARDDVKADMTAVVARKKAVVDGMADLFLGIFADTKVELIRG  117
             +VAH  R A ++G+GA   + + +K D+ +V  RK  +VDG+ D+ LG F +T VELI G
Sbjct  61    NVAHDLRSAQSYGLGAYVQSNNSLKVDLASVKRRKVEMVDGLVDMHLGKFRETGVELISG  120

Query  118   HGELAGPKTVRVN-GRLLTGNTVLINTGSKAFVDTKIPGLVDAKPLTHVELLNLTTLPSH  176
              G   GPKT++++ GRLLT +T++INTGS+A +D+ I GLV+AKPLTH+++L+   LP+H
Sbjct  121   EGRFVGPKTIQIDVGRLLTADTIVINTGSRATIDSSIKGLVEAKPLTHIDILDSEQLPAH  180

Query  177   LIILGGGYVGLEFAQAFVRFGAQVTVVERNSRVLKNEDDDVVASLTAILAREGVQFLTST  236
             L+I+GGGYV LEFAQAF RFG++VTV+E N ++LKNED+DVV +L  +L  EG++F+TS
Sbjct  181   LVIIGGGYVALEFAQAFKRFGSEVTVLEHNEQILKNEDEDVVNALLGVLKEEGIRFITSA  240

Query  237   SVARVSGTSGTEVVLTLSGPGP--AEIRGSHLLVATGRTPNTSGIGLVEAGIKLSNTGHV  294
              V  V G SG  V L++  P      I+ SHL+VA GRTPNTSGIGL + G+KL+  GHV
Sbjct  241   RVTEVQGISGQGVQLSVELPQGELQHIKSSHLMVAAGRTPNTSGIGLEKIGVKLTAIGHV  300

Query  295   AVDEQLGSSVPGVFAAGDCAGSPLFTHMGWDDYRIILASLTGSPREGGKTTRQVPSTLFT  354
              VDEQL ++  GVFA GDCAGSP FTH+G+DD+RI+ + L G+P+  G + RQVPSTLFT
```

```
Sbjct  301   QVDEQLRTTADGVFAVGDCAGSPHFTHIGFDDFRIVYSYLIGTPKTKGTSGRQVPSTLFT  360

Query  355   SPELAHVGLREEEAKAKGIPYRLTKAPMAAFLRTRALGETDGFAKALVEADGDRVLGFTA  414
             SPELAHVGL E EAKAKGI YRL K PMAAFLRTR LG+T GFAKAL+EADG+ +LGFTA
Sbjct  361   SPELAHVGLHEHEAKAKGIKYRLAKLPMAAFLRTRTLGDTRGFAKALIEADGNGILGFTA  420

Query  415   LGPSAGELLPAVQLVMKLGLSYKELVDLVLVHPTMCEGLVDLFRSVPPR  463
             LGPSAGE+LP VQL MKLG+ Y+E+  LV+ HPTMCEGL+ LF  V PR
Sbjct  421   LGPSAGEMLPVVQLAMKLGVGYQEIAGLVITHPTMCEGLLGLFSMVQPR  469
```

FAD/NAD(P)-binding domain-containing protein [Coniochaeta ligniaria NRRL 30616]

Sequence ID: **OIW30451.1** Length: 464 Number of Matches: 1

Range 1: 1 to 464

Score	Expect	Method	Identities	Positives	Gaps	Frame
574 bits(1479)	0.0()	Compositional matrix adjust.	280/464(60%)	355/464(76%)	4/464(0%)	

```
Query  1     MASPEQFDFVALGAGEPAKLLAWDLSSNHGMKCAVIEHGPIAGSCPTVACMPSKTIIHSA  60
             M+SPE FD V +G+GE  K  AW L S +G KCA+IE   + G+CP +AC+PSK  IHSA
Sbjct  1     MSSPEYFDCVIIGSGEAGKYTAWTLGSKYGKKCAIIERKYLGGACPNIACLPSKNFIHSA  60

Query  61    HVAHLARQAPAFGIG---AARDDVKADMTAVVARKKAVVDGMADLFLGIFADTKVELIRG  117
              VA+  RQA ++G+G   A  DDVK DMT    RK A+VDG+ DL +G F     E+I G
Sbjct  61    SVAYNFRQAASYGLGSFVAKADDVKVDMTVPGHRKVAMVDGLMDLHVGNFRKANTEVILG  120

Query  118   HGELAGPKTVRVNGRLLTGNTVLINTGSKAFVDTKIPGLVDAKPLTHVELLNLTTLPSHL  177
              G+  GPKT+ V GR+LTG+ +++NTG++A VD+ IPGLV++KPLTHVE+L+L  LPSHL
Sbjct  121   EGKFIGPKTIAVAGRMLTGDAIIVNTGTRALVDSTIPGLVESKPLTHVEILDLEVLPSHL  180

Query  178   IILGGGYVGLEFAQAFVRFGAQVTVVERNSRVLKNEDDDVVASLTAILAREGVQFLTSTS  237
             IILGGGYVGLEFAQA+ RFG+QVTV+E++ RVL  ED+D+V +L  I+  EGV+ +TS
Sbjct  181   IILGGGYVGLEFAQAYRRFGSQVTVIEKHERVLGKEDEDIVEALVGIITGEGVRIITSAK  240

Query  238   VARVSGTSGTEVVLTLSGPGP-AEIRGSHLLVATGRTPNTSGIGLVEAGIKLSNTGHVAV  296
             + +VSG SG  V ++++  G  ++I GSH+LVATGR PNT  +GL EAGI L+ + HV V
Sbjct  241   IDKVSGASGDSVQVSVNVAGSISKITGSHILVATGRVPNTENMGLEEAGINLTRSKHVLV  300

Query  297   DEQLGSSVPGVFAAGDCAGSPLFTHMGWDDYRIILASLTGSPREGGKTTRQVPSTLFTSP  356
             DEQL ++  GVFA GDCAGSP FTH+ +DD+R++L++LTG PR GG T RQVPS LFTS
Sbjct  301   DEQLRTTADGVFAVGDCAGSPYFTHISFDDFRVVLSNLTGCPRPGGTTDRQVPSVLFTSH  360

Query  357   ELAHVGLREEEAKAKGIPYRLTKAPMAAFLRTRALGETDGFAKALVEADGDRVLGFTALG  416
             ELAHVGLRE EA+ +GI YRL K PMAAFLRTR LG++ GFAK LVEA+GD++LGFTALG
Sbjct  361   ELAHVGLREHEAQDRGIKYRLGKMPMAAFLRTRTLGDSRGFAKVLVEAEGDKILGFTALG  420

Query  417   PSAGELLPAVQLVMKLGLSYKELVDLVLVHPTMCEGLVDLFRSV  460
              +AGELLP VQL MKLG+SYKE+ +LV+VHPTM EGLV LF SV
Sbjct  421   LAAGELLPVVQLAMKLGISYKEVSNLVIVHPTMGEGLVGLFNSV  464
```

hypothetical protein GE09DRAFT_1064680 [Coniochaeta sp. 2T2.1]

Sequence ID: **KAB5527974.1** Length: 464 Number of Matches: 1

Range 1: 1 to 464

Score	Expect	Method	Identities	Positives	Gaps	Frame
565 bits(1455)	0.0()	Compositional matrix adjust.	277/464(60%)	348/464(75%)	4/464(0%)	

```
Query  1     MASPEQFDFVALGAGEPAKLLAWDLSSNHGMKCAVIEHGPIAGSCPTVACMPSKTIIHSA  60
             MA P+ +D + +G GE  K + W L S HG KCA++E   + G+CP +AC+PSK  IHSA
Sbjct  1     MAQPQHYDVLVIGTGEGGKYVTWTLGSKHGKKCALVERRYLGGACPNIACLPSKNFIHSA  60

Query  61    HVAHLARQAPAFGIG---AARDDVKADMTAVVARKKAVVDGMADLFLGIFADTKVELIRG  117
              VA+  RQA ++G+G   A  D +K +MT    RK  +V G+ DL  G+F     E+I G
Sbjct  61    SVAYDVRQAASYGLGSFVANADGLKVNMTGPEQRKLDMVAGLKDLHEGLFQGANNEVIHG  120

Query  118   HGELAGPKTVRVNGRLLTGNTVLINTGSKAFVDTKIPGLVDAKPLTHVELLNLTTLPSHL  177
               +  GPKTV V GRLLTG+ V+INTG++AFVD+ +PGL ++KPLTHVE+L+L  LPSHL
Sbjct  121   EAKFIGPKTVEVGGRLLTGDAVVINTGTRAFVDSSVPGLAESKPLTHVEILDLDVLPSHL  180

Query  178   IILGGGYVGLEFAQAFVRFGAQVTVVERNSRVLKNEDDDVVASLTAILAREGVQFLTSTS  237
             IILGGGYVGLEFAQA+ RFG+QVTV+E++ RVL NED DVV +L  IL  EGV+ +TS
Sbjct  181   IILGGGYVGLEFAQAYRRFGSQVTVIEKHERVLGNEDKDVVDALVDILTGEGVRTITSAK  240

Query  238   VARVSGTSGTEVVLTLSGPGP-AEIRGSHLLVATGRTPNTSGIGLVEAGIKLSNTGHVAV  296
             V  V+GTSG  V ++++  G  + I GSH+LVATGR PNT  IGL EAGIKL+ + HV V
Sbjct  241   VENVTGTSGASVEVSINAAGQTSTITGSHILVATGRVPNTDSIGLEEAGIKLTASKHVKV  300

Query  297   DEQLGSSVPGVFAAGDCAGSPLFTHMGWDDYRIILASLTGSPREGGKTTRQVPSTLFTSP  356
             DEQL +SV GVFA GDCAGSP FTH+G+DD+R++ +++ G+P+ GG   RQVPS LFTS
Sbjct  301   DEQLKTSVEGVFAVGDCAGSPYFTHIGFDDFRVVYSNIMGTPKPGGTADRQVPSVLFTSH  360

Query  357   ELAHVGLREEEAKAKGIPYRLTKAPMAAFLRTRALGETDGFAKALVEADGDRVLGFTALG  416
             ELAHVGLRE+EAKAKGI YRL K PMAAFLRTR  G++ GFAK LVE  G+++LGFTALG
Sbjct  361   ELAHVGLREQEAKAKGIKYRLGKVPMAAFLRTRTKGDSRGFAKVLVEETGEKILGFTALG  420

Query  417   PSAGELLPAVQLVMKLGLSYKELVDLVLVHPTMCEGLVDLFRSV  460
              SAGELLP VQL MKLG+SYKE+ DL++VHPTM EGLV LF S+
Sbjct  421   FSAGELLPVVQLAMKLGISYKEIADLIIVHPTMGEGLVGLFNSL  464
```

Taxonomy

Reports

- **Lineage**

Organism	Blast Name	Score	Number of Hits	Description
cellular organisms			153	
.leotiomyceta	ascomycetes		33	
..sordariomyceta	ascomycetes		19	
...Sordariomycetidae	ascomycetes		10	
....Sordariales	ascomycetes		3	
.....Madurella mycetomatis	ascomycetes	932	1	Madurella mycetomatis hits
.....Chaetomium globosum CBS 148.51	ascomycetes	721	2	Chaetomium globosum CBS 148.51 hits
....Phaeoacremonium minimum UCRPA7	ascomycetes	581	2	Phaeoacremonium minimum UCRPA7 hits
....Coniochaeta ligniaria NRRL 30616	ascomycetes	574	1	Coniochaeta ligniaria NRRL 30616 hits
....Coniochaeta sp. 2T2.1	ascomycetes	565	2	Coniochaeta sp. 2T2.1 hits
....Gaeumannomyces tritici R3-111a-1	ascomycetes	479	2	Gaeumannomyces tritici R3-111a-1 hits
...Helotiales sp. DMI_Dod_QoI	ascomycetes	508	1	Helotiales sp. DMI_Dod_QoI hits
...Hyaloscypha variabilis F	ascomycetes	481	2	Hyaloscypha variabilis F hits
...Chalara longipes BDJ	ascomycetes	476	1	Chalara longipes BDJ hits
...Hyaloscypha bicolor E	ascomycetes	476	2	Hyaloscypha bicolor E hits
...Glarea lozoyensis ATCC 20868	ascomycetes	468	2	Glarea lozoyensis ATCC 20868 hits
...Pezoloma ericae	ascomycetes	457	1	Pezoloma ericae hits
..Exophiala mesophila	ascomycetes	496	1	Exophiala mesophila hits
..Trematosphaeria pertusa	ascomycetes	488	2	Trematosphaeria pertusa hits
..Clohesyomyces aquaticus	ascomycetes	478	1	Clohesyomyces aquaticus hits
..Lophiostoma macrostomum CBS 122681	ascomycetes	471	1	Lophiostoma macrostomum CBS 122681 hits
..Cenococcum geophilum 1.58	ascomycetes	470	1	Cenococcum geophilum 1.58 hits
..Stagonospora sp. SRC1lsM3a	ascomycetes	468	1	Stagonospora sp. SRC1lsM3a hits
..Pyrenochaeta sp. DS3sAY3a	ascomycetes	464	1	Pyrenochaeta sp. DS3sAY3a hits
..Ophiobolus disseminans	ascomycetes	461	1	Ophiobolus disseminans hits
..Rhizodiscina lignyota	ascomycetes	452	1	Rhizodiscina lignyota hits
..Zasmidium cellare ATCC 36951	ascomycetes	426	2	Zasmidium cellare ATCC 36951 hits
..Exserohilum turcica Et28A	ascomycetes	411	2	Exserohilum turcica Et28A hits
.Planctomyces sp. SH-PL62	planctomycetes	476	2	Planctomyces sp. SH-PL62 hits
.Paludisphaera sp. JC665	bacteria	473	1	Paludisphaera sp. JC665 hits
.Granulicella tundricola MP5ACTX9	bacteria	470	1	Granulicella tundricola MP5ACTX9 hits
.Granulicella tundricola	bacteria	469	1	Granulicella tundricola hits
.Planctomycetes bacterium EIP	bacteria	468	4	Planctomycetes bacterium EIP hits
.Gemmata massiliana	bacteria	466	4	Gemmata massiliana hits
.Gemmata sp. SH-PL17	bacteria	464	2	Gemmata sp. SH-PL17 hits
.Terrimicrobium sacchariphilum	verrucomicrobia	461	2	Terrimicrobium sacchariphilum hits
.Silvibacterium bohemicum	bacteria	461	2	Silvibacterium bohemicum hits
.Lacipirellula parvula	bacteria	456	2	Lacipirellula parvula hits
.Zavarzinella formosa	bacteria	456	1	Zavarzinella formosa hits
.Edaphobacter aggregans	bacteria	453	2	Edaphobacter aggregans hits
.Singulisphaera sp. GP187	bacteria	452	6	Singulisphaera sp. GP187 hits
.Verrucomicrobia bacterium	verrucomicrobia	453	1	Verrucomicrobia bacterium hits
.Singulisphaera acidiphila	bacteria	448	1	Singulisphaera acidiphila hits
.Singulisphaera acidiphila DSM 18658	bacteria	448	1	Singulisphaera acidiphila DSM 18658 hits
.Paludisphaera borealis	bacteria	444	2	Paludisphaera borealis hits
.Granulicella mallensis	bacteria	442	2	Granulicella mallensis hits
.Paraburkholderia sp. RP-4-7	b-proteobacteria	442	2	Paraburkholderia sp. RP-4-7 hits
.Granulicella sp. WH15	bacteria	440	2	Granulicella sp. WH15 hits
.Granulicella sp. S190	bacteria	438	1	Granulicella sp. S190 hits
.Acidobacteriaceae bacterium KBS 146	bacteria	437	1	Acidobacteriaceae bacterium KBS 146 hits
.Frigoriglobus tundricola	bacteria	437	2	Frigoriglobus tundricola hits

.Edaphobacter sp. 12200R-103	bacteria	437	2	Edaphobacter sp. 12200R-103 hits
.Granulicella sp. GAS466	bacteria	434	2	Granulicella sp. GAS466 hits
.Granulicella sp. L60	bacteria	434	1	Granulicella sp. L60 hits
.Gemmata obscuriglobus	bacteria	432	3	Gemmata obscuriglobus hits
.Gemmata obscuriglobus UQM 2246	bacteria	432	1	Gemmata obscuriglobus UQM 2246 hits
.Terriglobus roseus	bacteria	426	2	Terriglobus roseus hits
.Acidobacterium sp. 4Y35	bacteria	426	2	Acidobacterium sp. 4Y35 hits
.Methylobacterium sp. 174MFSha1.1	a-proteobacteria	416	2	Methylobacterium sp. 174MFSha1.1 hits
.Acetobacter nitrogenifigens	a-proteobacteria	416	1	Acetobacter nitrogenifigens hits
.Acetobacter nitrogenifigens DSM 23921 = LMG 23498	a-proteobacteria	416	1	Acetobacter nitrogenifigens DSM 23921 = LMG 23498 hits
.Capsulimonas corticalis	bacteria	415	2	Capsulimonas corticalis hits
.Acidisarcina polymorpha	bacteria	412	2	Acidisarcina polymorpha hits
.Methylobacterium sp. 6HR-1	a-proteobacteria	411	2	Methylobacterium sp. 6HR-1 hits
.Bryocella elongata	bacteria	411	2	Bryocella elongata hits
.Mesorhizobium ciceri	a-proteobacteria	410	1	Mesorhizobium ciceri hits
.Methylobacterium sp. 17Sr1-43	a-proteobacteria	408	2	Methylobacterium sp. 17Sr1-43 hits
.Methylobacterium variabile	a-proteobacteria	408	2	Methylobacterium variabile hits
.Rhodanobacter fulvus	g-proteobacteria	407	1	Rhodanobacter fulvus hits
.Rhodanobacter fulvus Jip2	g-proteobacteria	407	1	Rhodanobacter fulvus Jip2 hits
.Granulicella sibirica	bacteria	406	4	Granulicella sibirica hits
.Pseudaminobacter manganicus	a-proteobacteria	406	2	Pseudaminobacter manganicus hits
.Acidobacteria bacterium	bacteria	406	3	Acidobacteria bacterium hits
.Aquisphaera sp. JC669	bacteria	405	1	Aquisphaera sp. JC669 hits
.Fimbriiglobus ruber	bacteria	405	2	Fimbriiglobus ruber hits
.Rhodanobacter sp. OR444	g-proteobacteria	404	1	Rhodanobacter sp. OR444 hits
.Rhodanobacter sp.	g-proteobacteria	404	1	Rhodanobacter sp. hits
.Methylobacterium tarhaniae	a-proteobacteria	403	2	Methylobacterium tarhaniae hits
.Rhodanobacter denitrificans	g-proteobacteria	403	2	Rhodanobacter denitrificans hits
.Opitutus sp. ER46	verrucomicrobia	402	2	Opitutus sp. ER46 hits
.Mesorhizobium sp. B2-3-3	a-proteobacteria	400	1	Mesorhizobium sp. B2-3-3 hits
.Acidobacteria bacterium Pan2503	bacteria	398	1	Acidobacteria bacterium Pan2503 hits
.Mesorhizobium sp. B2-3-5	a-proteobacteria	400	2	Mesorhizobium sp. B2-3-5 hits
.Mesorhizobium sp. B2-4-15	a-proteobacteria	399	2	Mesorhizobium sp. B2-4-15 hits
.Rhodanobacter thiooxydans	g-proteobacteria	398	2	Rhodanobacter thiooxydans hits
.Rhodanobacter thiooxydans LCS2	g-proteobacteria	398	1	Rhodanobacter thiooxydans LCS2 hits
.Duganella sp. DN04	b-proteobacteria	396	2	Duganella sp. DN04 hits
.Pseudomonas acidophila	g-proteobacteria	396	1	Pseudomonas acidophila hits
.Paraburkholderia caledonica	b-proteobacteria	394	2	Paraburkholderia caledonica hits
.Mesorhizobium sp. B4-1-4	a-proteobacteria	394	2	Mesorhizobium sp. B4-1-4 hits
.Pirellula sp.	bacteria	392	1	Pirellula sp. hits
.Burkholderia sp. WSM2230	b-proteobacteria	392	1	Burkholderia sp. WSM2230 hits
.Mesorhizobium sp. B2-4-12	a-proteobacteria	391	2	Mesorhizobium sp. B2-4-12 hits
.Methylobacterium crusticola	a-proteobacteria	391	1	Methylobacterium crusticola hits
.Mesorhizobium sp. B2-4-17	a-proteobacteria	390	2	Mesorhizobium sp. B2-4-17 hits

Organism

Description	Score	E value	Accession
Madurella mycetomatis [ascomycetes]			
**Leghemoglobin reductase [Madurella mycetomatis]**	932	0.0	KXX77420
Chaetomium globosum CBS 148.51 [ascomycetes]			

Description	Score	E value	Accession
hypothetical protein CHGG_00138 [Chaetomium globosum CBS 148.51]	721	0.0	XP_001219359
hypothetical protein CHGG_00138 [Chaetomium globosum CBS 148.51]	721	0.0	EAQ91903
Phaeoacremonium minimum UCRPA7 [ascomycetes]			
putative dihydrolipoyl dehydrogenase protein [Phaeoacremonium minimum UCRPA7]	581	0.0	XP_007915022
putative dihydrolipoyl dehydrogenase protein [Phaeoacremonium minimum UCRPA7]	581	0.0	EOO00199
Coniochaeta ligniaria NRRL 30616 [ascomycetes]			
FAD/NAD(P)-binding domain-containing protein [Coniochaeta ligniaria NRRL 30616]	574	0.0	OIW30451
Coniochaeta sp. 2T2.1 [ascomycetes]			
hypothetical protein GE09DRAFT_1064680 [Coniochaeta sp. 2T2.1]	565	0.0	KAB5527974
hypothetical protein GE09DRAFT_15414 [Coniochaeta sp. 2T2.1]	555	0.0	KAB5585116
Helotiales sp. DMI_Dod_QoI [ascomycetes]			
hypothetical protein EG329_003274 [Helotiales sp. DMI_Dod_QoI]	508	1e-174	KAE8442493
Exophiala mesophila [ascomycetes]			
hypothetical protein B0A52_07332 [Exophiala mesophila]	496	3e-170	RVX68329
Trematosphaeria pertusa [ascomycetes]			
FAD-dependent pyridine nucleotide-disulfide oxidoreductase [Trematosphaeria pertusa]	488	8e-167	XP_033688688
FAD-dependent pyridine nucleotide-disulfide oxidoreductase [Trematosphaeria pertusa]	488	8e-167	KAF2253684
Hyaloscypha variabilis F [ascomycetes]			
Dihydrolipoyl dehydrogenase [Hyaloscypha variabilis F]	481	6e-164	PMD29251
FAD/NAD(P)-binding domain-containing protein [Hyaloscypha variabilis F]	433	3e-146	PMD31504
Gaeumannomyces tritici R3-111a-1 [ascomycetes]			
hypothetical protein GGTG_11046 [Gaeumannomyces tritici R3-111a-1]	479	3e-163	XP_009227190
hypothetical protein GGTG_11046 [Gaeumannomyces tritici R3-111a-1]	479	3e-163	EJT71793
Clohesyomyces aquaticus [ascomycetes]			
FAD-dependent pyridine nucleotide-disulfide oxidoreductase [Clohesyomyces aquaticus]	478	5e-163	ORY16189
Chalara longipes BDJ [ascomycetes]			
FAD/NAD(P)-binding domain-containing protein [Chalara longipes BDJ]	476	3e-162	KAE9375044
Planctomyces sp. SH-PL62 [planctomycetes]			
FAD-dependent oxidoreductase [Planctomyces sp. SH-PL62]	476	3e-162	WP_068422479
Mercuric reductase [Planctomyces sp. SH-PL62]	476	3e-162	AMV40164
Hyaloscypha bicolor E [ascomycetes]			
Dihydrolipoyl dehydrogenase [Hyaloscypha bicolor E]	476	6e-162	XP_024741558
Dihydrolipoyl dehydrogenase [Hyaloscypha bicolor E]	476	6e-162	PMD64654
Paludisphaera sp. JC665 [bacteria]			
FAD-dependent oxidoreductase [Paludisphaera sp. JC665]	473	3e-161	WP_165070541
Lophiostoma macrostomum CBS 122681 [ascomycetes]			
FAD-dependent pyridine nucleotide-disulfide oxidoreductase [Lophiostoma macrostomum CBS 122681]	471	2e-160	KAF2658962
Cenococcum geophilum 1.58 [ascomycetes]			
FAD-dependent pyridine nucleotide-disulfide oxidoreductase [Cenococcum geophilum 1.58]	470	1e-159	OCK89743
Granulicella tundricola MP5ACTX9 [bacteria]			
FAD-dependent pyridine nucleotide-disulfide oxidoreductase [Granulicella tundricola MP5ACTX9]	470	2e-159	ADW69689
Granulicella tundricola [bacteria]			
FAD-dependent oxidoreductase [Granulicella tundricola]	469	2e-159	WP_041597111
Planctomycetes bacterium EIP [bacteria]			
FAD-dependent oxidoreductase [Planctomycetes bacterium EIP]	468	5e-159	WP_145272511
Mercuric reductase [Planctomycetes bacterium EIP]	468	5e-159	QDV36331
FAD-dependent oxidoreductase [Planctomycetes bacterium EIP]	419	5e-140	WP_145272489
Mercuric reductase [Planctomycetes bacterium EIP]	419	5e-140	QDV36319
Glarea lozoyensis ATCC 20868 [ascomycetes]			
FAD/NAD(P)-binding protein [Glarea lozoyensis ATCC 20868]	468	7e-159	XP_008075901
FAD/NAD(P)-binding protein [Glarea lozoyensis ATCC 20868]	468	7e-159	EPE36586

Description	Score	E value	Accession
Stagonospora sp. SRC1lsM3a [ascomycetes]			
Dihydrolipoyl dehydrogenase [Stagonospora sp. SRC1lsM3a]	468	8e-159	OAK94831
Gemmata massiliana [bacteria]			
FAD-dependent oxidoreductase [Gemmata massiliana]	466	5e-158	WP_162672821
mercuric reductase : Pyruvate/2-oxoglutarate dehydrogenase complex, dihydrolipoamide dehydrogenase component OS=Singulisphaera acidiphila (strain ATCC BAA-1392 / DSM 18658 / VKM B-2454 / MOB10) GN=Sinac_3751 PE=4 SV=1: Pyr_redox_2: Pyr_redox: Pyr_redox_dim [Gemmata massiliana]	466	5e-158	VTS02724
FAD-dependent oxidoreductase [Gemmata massiliana]	422	5e-141	WP_162670453
mercuric reductase : Pyruvate/2-oxoglutarate dehydrogenase complex, dihydrolipoamide dehydrogenase component OS=Singulisphaera acidiphila (strain ATCC BAA-1392 / DSM 18658 / VKM B-2454 / MOB10) GN=Sinac_3751 PE=4 SV=1: Pyr_redox_2: Pyr_redox: Pyr_redox_dim [Gemmata massiliana]	422	5e-141	VTR96120
Gemmata sp. SH-PL17 [bacteria]			
FAD-dependent oxidoreductase [Gemmata sp. SH-PL17]	464	1e-157	WP_082838148
Mercuric reductase [Gemmata sp. SH-PL17]	464	1e-157	AMV22920
Pyrenochaeta sp. DS3sAY3a [ascomycetes]			
Dihydrolipoyl dehydrogenase [Pyrenochaeta sp. DS3sAY3a]	464	3e-157	OAL51325
Ophiobolus disseminans [ascomycetes]			
Dihydrolipoyl dehydrogenase [Ophiobolus disseminans]	461	2e-156	KAF2824058
Terrimicrobium sacchariphilum [verrucomicrobia]			
FAD-dependent oxidoreductase [Terrimicrobium sacchariphilum]	461	3e-156	WP_075080549
pyruvate/2-oxoglutarate dehydrogenase complex [Terrimicrobium sacchariphilum]	460	8e-156	GAT31921
Silvibacterium bohemicum [bacteria]			
FAD-dependent oxidoreductase [Silvibacterium bohemicum]	461	3e-156	WP_050061725
pyruvate/2-oxoglutarate dehydrogenase complex dihydrolipoamide dehydrogenase (E3) component [Silvibacterium bohemicum]	461	3e-156	MBB6145150
Pezoloma ericae [ascomycetes]			
Dihydrolipoyl dehydrogenase [Pezoloma ericae]	457	2e-154	PMD16242
Lacipirellula parvula [bacteria]			
FAD-dependent oxidoreductase [Lacipirellula parvula]	456	3e-154	WP_152100060
hypothetical protein PLANPX_4106 [Lacipirellula parvula]	456	3e-154	BBO34494
Zavarzinella formosa [bacteria]			
FAD-dependent oxidoreductase [Zavarzinella formosa]	456	3e-154	WP_020474773
Edaphobacter aggregans [bacteria]			
FAD-dependent oxidoreductase [Edaphobacter aggregans]	453	3e-153	WP_125484885
pyruvate/2-oxoglutarate dehydrogenase complex dihydrolipoamide dehydrogenase (E3) component [Edaphobacter aggregans]	453	3e-153	RSL16250
Singulisphaera sp. GP187 [bacteria]			
FAD-dependent oxidoreductase [Singulisphaera sp. GP187]	452	4e-153	WP_074308700
Pyruvate/2-oxoglutarate dehydrogenase complex, dihydrolipoamide dehydrogenase (E3) component [Singulisphaera sp. GP187]	452	4e-153	SIO33452
Pyruvate/2-oxoglutarate dehydrogenase complex, dihydrolipoamide dehydrogenase (E3) component [Singulisphaera sp. GP187]	445	1e-149	SIO66840
FAD-dependent oxidoreductase [Singulisphaera sp. GP187]	444	1e-149	WP_074317373
FAD-dependent oxidoreductase [Singulisphaera sp. GP187]	429	1e-143	WP_074316341
Pyruvate/2-oxoglutarate dehydrogenase complex, dihydrolipoamide dehydrogenase (E3) component [Singulisphaera sp. GP187]	429	1e-143	SIO65342
Verrucomicrobia bacterium [verrucomicrobia]			
mercuric reductase [Verrucomicrobia bacterium]	453	6e-153	PYK02771
Rhizodiscina lignyota [ascomycetes]			
FAD-dependent pyridine nucleotide-disulfide oxidoreductase [Rhizodiscina lignyota]	452	2e-152	KAF2093634
Singulisphaera acidiphila [bacteria]			
FAD-dependent oxidoreductase [Singulisphaera acidiphila]	448	2e-151	WP_015247128
Singulisphaera acidiphila DSM 18658 [bacteria]			
pyruvate/2-oxoglutarate dehydrogenase complex, dihydrolipoamide dehydrogenase component [Singulisphaera acidiphila DSM 18658]	448	2e-151	AGA27989

Description	Score	E value	Accession
Paludisphaera borealis [bacteria]			
putative pyridine nucleotide-disulfide oxidoreductase RclA [Paludisphaera borealis]	444	2e-149	APW61714
FAD-dependent oxidoreductase [Paludisphaera borealis]	441	2e-148	WP_076350975
Granulicella mallensis [bacteria]			
FAD-dependent oxidoreductase [Granulicella mallensis]	442	1e-148	WP_184254370
pyruvate/2-oxoglutarate dehydrogenase complex dihydrolipoamide dehydrogenase (E3) component [Granulicella mallensis]	442	2e-148	MBB5063034
Paraburkholderia sp. RP-4-7 [b-proteobacteria]			
FAD-dependent oxidoreductase [Paraburkholderia sp. RP-4-7]	442	3e-148	WP_169491108
FAD-dependent oxidoreductase [Paraburkholderia sp. RP-4-7]	442	3e-148	NMM04363
Granulicella sp. WH15 [bacteria]			
FAD-dependent oxidoreductase [Granulicella sp. WH15]	440	5e-148	WP_162539072
mercuric reductase [Granulicella sp. WH15]	440	5e-148	QHN04713
Granulicella sp. S190 [bacteria]			
FAD-dependent oxidoreductase [Granulicella sp. S190]	438	2e-147	WP_158943510
Acidobacteria bacterium KBS 146 [bacteria]			
FAD-dependent oxidoreductase [Acidobacteriaceae bacterium KBS 146]	437	5e-147	WP_026388560
Frigoriglobus tundricola [bacteria]			
FAD-dependent oxidoreductase [Frigoriglobus tundricola]	437	6e-147	WP_171470804
PF00070 family, FAD-dependent NAD(P)-disulfide oxidoreductase [Frigoriglobus tundricola]	437	6e-147	QJW94903
Edaphobacter sp. 12200R-103 [bacteria]			
FAD-dependent oxidoreductase [Edaphobacter sp. 12200R-103]	437	7e-147	WP_162402250
FAD-dependent oxidoreductase [Edaphobacter sp. 12200R-103]	437	7e-147	QHS52141
Granulicella sp. GAS466 [bacteria]			
FAD-dependent oxidoreductase [Granulicella sp. GAS466]	434	2e-145	WP_123489827
pyruvate/2-oxoglutarate dehydrogenase complex dihydrolipoamide dehydrogenase (E3) component [Granulicella sp. GAS466]	434	2e-145	ROP69591
Granulicella sp. L60 [bacteria]			
FAD-dependent oxidoreductase [Granulicella sp. L60]	434	2e-145	WP_158790451
Gemmata obscuriglobus [bacteria]			
FAD-dependent oxidoreductase [Gemmata obscuriglobus]	432	6e-145	WP_010053189
mercuric reductase [Gemmata obscuriglobus]	432	6e-145	AWM36035
Mercuric reductase [Gemmata obscuriglobus]	432	6e-145	QEG31390
Gemmata obscuriglobus UQM 2246 [bacteria]			
mercuric reductase : Pyruvate/2-oxoglutarate dehydrogenase complex, dihydrolipoamide dehydrogenase component OS=Singulisphaera acidiphila (strain ATCC BAA-1392 / DSM 18658 / VKM B-2454 / MOB10) GN=Sinac_3751 PE=4 SV=1: Pyr_redox_2: Pyr_redox: Pyr_redox_dim [Gemmata obscuriglobus UQM 2246]	432	6e-145	VTS10730
Zasmidium cellare ATCC 36951 [ascomycetes]			
uncharacterized protein M409DRAFT_64983 [Zasmidium cellare ATCC 36951]	426	6e-143	XP_033670150
hypothetical protein M409DRAFT_64983 [Zasmidium cellare ATCC 36951]	426	6e-143	KAF2169261
Terriglobus roseus [bacteria]			
Pyruvate/2-oxoglutarate dehydrogenase complex, dihydrolipoamide dehydrogenase (E3) component [Terriglobus roseus]	426	2e-142	SDF98400
FAD-dependent oxidoreductase [Terriglobus roseus]	426	3e-142	WP_156785218
Acidobacterium sp. 4Y35 [bacteria]			
FAD-dependent oxidoreductase [Acidobacterium sp. 4Y35]	426	2e-142	WP_186745562
FAD-dependent oxidoreductase [Acidobacterium sp. 4Y35]	426	2e-142	QNI33859
Methylobacterium sp. 174MFSha1.1 [a-proteobacteria]			
mercuric reductase [Methylobacterium sp. 174MFSha1.1]	416	8e-139	WP_093569215
Pyruvate/2-oxoglutarate dehydrogenase complex, dihydrolipoamide dehydrogenase (E3) component [Methylobacterium sp. 174MFSha1.1]	416	8e-139	SFV05526
Acetobacter nitrogenifigens [a-proteobacteria]			
FAD-dependent oxidoreductase [Acetobacter nitrogenifigens]	416	2e-138	WP_026397664

Description	Score	E value	Accession
Acetobacter nitrogenifigens DSM 23921 = LMG 23498 [a-proteobacteria]			
pyridine nucleotide-disulfide oxidoreductase [Acetobacter nitrogenifigens DSM 23921 = LMG 23498]	416	2e-138	GEN59903
Capsulimonas corticalis [bacteria]			
FAD-dependent oxidoreductase [Capsulimonas corticalis]	415	3e-138	WP_119321024
Mercuric reductase [Capsulimonas corticalis]	415	3e-138	GCE53596
Acidisarcina polymorpha [bacteria]			
FAD-dependent oxidoreductase [Acidisarcina polymorpha]	412	6e-137	WP_114205464
PF00070 family, FAD-dependent NAD(P)-disulfide oxidoreductase [Acidisarcina polymorpha]	412	6e-137	AXC09672
Methylobacterium sp. 6HR-1 [a-proteobacteria]			
mercuric reductase [Methylobacterium sp. 6HR-1]	411	9e-137	WP_135416742
mercuric reductase [Methylobacterium sp. 6HR-1]	411	9e-137	TGD97672
Bryocella elongata [bacteria]			
Pyruvate/2-oxoglutarate dehydrogenase complex, dihydrolipoamide dehydrogenase (E3) component [Bryocella elongata]	411	1e-136	SEG03116
FAD-dependent oxidoreductase [Bryocella elongata]	410	2e-136	WP_103932789
Exserohilum turcica Et28A [ascomycetes]			
uncharacterized protein SETTUDRAFT_159632 [Exserohilum turcica Et28A]	411	2e-136	XP_008023056
hypothetical protein SETTUDRAFT_159632 [Exserohilum turcica Et28A]	411	2e-136	EOA89225
Mesorhizobium ciceri [a-proteobacteria]			
mercuric reductase [Mesorhizobium ciceri]	410	3e-136	WP_027035822
Methylobacterium sp. 17Sr1-43 [a-proteobacteria]			
mercuric reductase [Methylobacterium sp. 17Sr1-43]	408	1e-135	WP_109952220
mercuric reductase [Methylobacterium sp. 17Sr1-43]	408	1e-135	AWN37141
Methylobacterium variabile [a-proteobacteria]			
mercuric reductase [Methylobacterium variabile]	408	2e-135	WP_048443666
mercuric reductase [Methylobacterium variabile]	408	2e-135	KMO40420
Rhodanobacter fulvus [g-proteobacteria]			
mercuric reductase [Rhodanobacter fulvus]	407	6e-135	WP_007081217
Rhodanobacter fulvus Jip2 [g-proteobacteria]			
dihydrolipoyl dehydrogenase [Rhodanobacter fulvus Jip2]	407	6e-135	EIL89884
Granulicella sibirica [bacteria]			
FAD-dependent oxidoreductase [Granulicella sibirica]	406	7e-135	WP_128912808
PF00070 family, FAD-dependent NAD(P)-disulfide oxidoreductase [Granulicella sibirica]	406	7e-135	RXH58894
FAD-dependent oxidoreductase [Granulicella sibirica]	405	2e-134	WP_128914270
PF00070 family, FAD-dependent NAD(P)-disulfide oxidoreductase [Granulicella sibirica]	405	2e-134	RXH54491
Pseudaminobacter manganicus [a-proteobacteria]			
mercuric reductase [Pseudaminobacter manganicus]	406	7e-135	WP_080921142
mercuric reductase [Pseudaminobacter manganicus]	406	7e-135	OQM73900
Acidobacteria bacterium [bacteria]			
mercuric reductase [Acidobacteria bacterium]	406	1e-134	PYU19249
mercuric reductase [Acidobacteria bacterium]	405	1e-134	PYQ03069
mercuric reductase [Acidobacteria bacterium]	399	1e-131	PYX73050
Aquisphaera sp. JC669 [bacteria]			
FAD-dependent oxidoreductase [Aquisphaera sp. JC669]	405	1e-134	WP_165224774
Fimbriiglobus ruber [bacteria]			
FAD-dependent oxidoreductase [Fimbriiglobus ruber]	405	3e-134	WP_088259766
mercuric reductase [Fimbriiglobus ruber]	405	3e-134	OWK36821
Rhodanobacter sp. OR444 [g-proteobacteria]			
mercuric reductase [Rhodanobacter sp. OR444]	404	8e-134	WP_027491238
Rhodanobacter sp. [g-proteobacteria]			
mercuric reductase [Rhodanobacter sp.]	404	8e-134	TAN19602
Methylobacterium tarhaniae [a-proteobacteria]			

Description	Score	E value	Accession
mercuric reductase [Methylobacterium tarhaniae]	403	1e-133	WP_048454291
mercuric reductase [Methylobacterium tarhaniae]	403	1e-133	KMO30360
Rhodanobacter denitrificans [g-proteobacteria]			
mercuric reductase [Rhodanobacter denitrificans]	403	2e-133	WP_114340465
mercuric reductase [Rhodanobacter denitrificans]	403	2e-133	RCS31689
Opitutus sp. ER46 [verrucomicrobia]			
FAD-dependent oxidoreductase [Opitutus sp. ER46]	402	6e-133	WP_107835729
mercuric reductase [Opitutus sp. ER46]	402	6e-133	PTX94332
Mesorhizobium sp. B2-3-3 [a-proteobacteria]			
mercuric reductase [Mesorhizobium sp. B2-3-3]	400	2e-132	TPN23623
Acidobacteria bacterium Pan2503 [bacteria]			
FAD-dependent oxidoreductase [Acidobacteria bacterium Pan2503]	398	2e-132	MBA0087722
Mesorhizobium sp. B2-3-5 [a-proteobacteria]			
FAD-dependent oxidoreductase [Mesorhizobium sp. B2-3-5]	400	4e-132	WP_140747153
mercuric reductase [Mesorhizobium sp. B2-3-5]	400	4e-132	TPM21527
Mesorhizobium sp. B2-4-15 [a-proteobacteria]			
FAD-dependent oxidoreductase [Mesorhizobium sp. B2-4-15]	399	8e-132	WP_141840360
mercuric reductase [Mesorhizobium sp. B2-4-15]	399	8e-132	TPK66823
Rhodanobacter thiooxydans [g-proteobacteria]			
mercuric reductase [Rhodanobacter thiooxydans]	398	2e-131	WP_008438212
mercuric reductase [Rhodanobacter thiooxydans]	398	2e-131	KZC21982
Rhodanobacter thiooxydans LCS2 [g-proteobacteria]			
dihydrolipoyl dehydrogenase [Rhodanobacter thiooxydans LCS2]	398	2e-131	EIL97526
Duganella sp. DN04 [b-proteobacteria]			
mercuric reductase [Duganella sp. DN04]	396	6e-131	WP_135202832
mercuric reductase [Duganella sp. DN04]	396	6e-131	TFW18649
Pseudomonas acidophila [g-proteobacteria]			
mercuric reductase [Pseudomonas acidophila]	396	9e-131	PCE23994
Paraburkholderia caledonica [b-proteobacteria]			
FAD-dependent oxidoreductase [Paraburkholderia caledonica]	394	4e-130	WP_087753710
mercuric reductase [Paraburkholderia caledonica]	392	3e-129	WP_020067768
Mesorhizobium sp. B4-1-4 [a-proteobacteria]			
FAD-dependent oxidoreductase [Mesorhizobium sp. B4-1-4]	394	9e-130	WP_140766259
mercuric reductase [Mesorhizobium sp. B4-1-4]	394	9e-130	TPI15652
Pirellula sp. [bacteria]			
mercuric reductase [Pirellula sp.]	392	2e-129	MBA4105228
Burkholderia sp. WSM2230 [b-proteobacteria]			
mercuric reductase [Burkholderia sp. WSM2230]	392	2e-129	WP_025598434
Mesorhizobium sp. B2-4-12 [a-proteobacteria]			
mercuric reductase [Mesorhizobium sp. B2-4-12]	391	7e-129	WP_140610964
mercuric reductase [Mesorhizobium sp. B2-4-12]	391	7e-129	TPK93458
Methylobacterium sp. MIMD6 [a-proteobacteria]			
FAD-dependent oxidoreductase [Methylobacterium crusticola]	391	8e-129	WP_128565516
Mesorhizobium sp. B2-4-17 [a-proteobacteria]			
FAD-dependent oxidoreductase [Mesorhizobium sp. B2-4-17]	390	1e-128	WP_140776343
mercuric reductase [Mesorhizobium sp. B2-4-17]	390	1e-128	TPK92515

Taxonomy

○

Taxonomy	Number of hits	Number of Organisms	Description
cellular organisms	153	90	

. leotiomyceta	33	23	
.. sordariomyceta	19	12	
... Sordariomycetidae	10	6	
.... Sordariales	3	2	
..... Madurella mycetomatis	1	1	**Madurella mycetomatis hits**
..... Chaetomium globosum CBS 148.51	2	1	**Chaetomium globosum CBS 148.51 hits**
.... Phaeoacremonium minimum UCRPA7	2	1	**Phaeoacremonium minimum UCRPA7 hits**
.... Coniochaeta	3	2	
..... Coniochaeta ligniaria NRRL 30616	1	1	**Coniochaeta ligniaria NRRL 30616 hits**
..... Coniochaeta sp. 2T2.1	2	1	**Coniochaeta sp. 2T2.1 hits**
.... Gaeumannomyces tritici R3-111a-1	2	1	**Gaeumannomyces tritici R3-111a-1 hits**
... Helotiales	9	6	
.... Helotiales sp. DMI_Dod_QoI	1	1	**Helotiales sp. DMI_Dod_QoI hits**
.... Hyaloscypha	4	2	
..... Hyaloscypha variabilis F	2	1	**Hyaloscypha variabilis F hits**
..... Hyaloscypha bicolor E	2	1	**Hyaloscypha bicolor E hits**
.... Chalara longipes BDJ	1	1	**Chalara longipes BDJ hits**
.... Glarea lozoyensis ATCC 20868	2	1	**Glarea lozoyensis ATCC 20868 hits**
.... Pezoloma ericae	1	1	**Pezoloma ericae hits**
.. Exophiala mesophila	1	1	**Exophiala mesophila hits**
.. Dothideomycetes	13	10	
... Pleosporomycetidae	11	9	
.... Pleosporales	9	7	
..... Massarineae	3	2	
...... Trematosphaeria pertusa	2	1	**Trematosphaeria pertusa hits**
...... Stagonospora sp. SRC1lsM3a	1	1	**Stagonospora sp. SRC1lsM3a hits**
..... Clohesyomyces aquaticus	1	1	**Clohesyomyces aquaticus hits**
..... Lophiostoma macrostomum CBS 122681	1	1	**Lophiostoma macrostomum CBS 122681 hits**
..... Pleosporineae	4	3	
...... Pyrenochaeta sp. DS3sAY3a	1	1	**Pyrenochaeta sp. DS3sAY3a hits**
...... Ophiobolus disseminans	1	1	**Ophiobolus disseminans hits**
...... Exserohilum turcica Et28A	2	1	**Exserohilum turcica Et28A hits**
.... Cenococcum geophilum 1.58	1	1	**Cenococcum geophilum 1.58 hits**
.... Rhizodiscina lignyota	1	1	**Rhizodiscina lignyota hits**
... Zasmidium cellare ATCC 36951	2	1	**Zasmidium cellare ATCC 36951 hits**
. Bacteria	120	67	
.. PVC group	41	20	
... Planctomycetes	36	17	
.... Planctomycetia	32	16	
..... Planctomyces sp. SH-PL62	2	1	**Planctomyces sp. SH-PL62 hits**
..... Isosphaeraceae	12	6	
...... Paludisphaera	3	2	
.... Paludisphaera sp. JC665	1	1	**Paludisphaera sp. JC665 hits**
.... Paludisphaera borealis	2	1	**Paludisphaera borealis hits**
...... Singulisphaera	8	3	
.... Singulisphaera sp. GP187	6	1	**Singulisphaera sp. GP187 hits**
.... Singulisphaera acidiphila	1	2	**Singulisphaera acidiphila hits**
.... Singulisphaera acidiphila DSM 18658	1	1	**Singulisphaera acidiphila DSM 18658 hits**
...... Aquisphaera sp. JC669	1	1	**Aquisphaera sp. JC669 hits**
..... Gemmataceae	15	7	
...... Gemmata	10	4	
.... Gemmata massiliana	4	1	**Gemmata massiliana hits**
.... Gemmata sp. SH-PL17	2	1	**Gemmata sp. SH-PL17 hits**

.... Gemmata obscuriglobus	3	2	Gemmata obscuriglobus hits
.... Gemmata obscuriglobus UQM 2246	1	1	Gemmata obscuriglobus UQM 2246 hits
...... Zavarzinella formosa	1	1	Zavarzinella formosa hits
...... Frigoriglobus tundricola	2	1	Frigoriglobus tundricola hits
...... Fimbriiglobus ruber	2	1	Fimbriiglobus ruber hits
..... Pirellulales	3	2	
...... Lacipirellula parvula	2	1	Lacipirellula parvula hits
...... Pirellula sp.	1	1	Pirellula sp. hits
.... Planctomycetes bacterium EIP	4	1	Planctomycetes bacterium EIP hits
... Verrucomicrobia	5	3	
.... Terrimicrobium sacchariphilum	2	1	Terrimicrobium sacchariphilum hits
.... Verrucomicrobia bacterium	1	1	Verrucomicrobia bacterium hits
.... Opitutus sp. ER46	2	1	Opitutus sp. ER46 hits
.. Acidobacteria	33	18	
... Acidobacteriia	30	17	
.... Acidobacteriaceae	29	16	
..... Granulicella	14	8	
...... Granulicella tundricola	1	2	Granulicella tundricola hits
.... Granulicella tundricola MP5ACTX9	1	1	Granulicella tundricola MP5ACTX9 hits
...... Granulicella mallensis	2	1	Granulicella mallensis hits
...... unclassified Granulicella	6	4	
.... Granulicella sp. WH15	2	1	Granulicella sp. WH15 hits
.... Granulicella sp. S190	1	1	Granulicella sp. S190 hits
.... Granulicella sp. GAS466	2	1	Granulicella sp. GAS466 hits
.... Granulicella sp. L60	1	1	Granulicella sp. L60 hits
...... Granulicella sibirica	4	1	Granulicella sibirica hits
..... Silvibacterium bohemicum	2	1	Silvibacterium bohemicum hits
..... Edaphobacter	4	2	
...... Edaphobacter aggregans	2	1	Edaphobacter aggregans hits
...... Edaphobacter sp. 12200R-103	2	1	Edaphobacter sp. 12200R-103 hits
..... Acidobacteriaceae bacterium KBS 146	1	1	Acidobacteriaceae bacterium KBS 146 hits
..... Terriglobus roseus	2	1	Terriglobus roseus hits
..... Acidobacterium sp. 4Y35	2	1	Acidobacterium sp. 4Y35 hits
..... Acidisarcina polymorpha	2	1	Acidisarcina polymorpha hits
..... Bryocella elongata	2	1	Bryocella elongata hits
.... Acidobacteria bacterium Pan2503	1	1	Acidobacteria bacterium Pan2503 hits
... Acidobacteria bacterium	3	1	Acidobacteria bacterium hits
.. Proteobacteria	44	28	
... Burkholderiales	7	4	
.... Burkholderiaceae	5	3	
..... Paraburkholderia	4	2	
...... Paraburkholderia sp. RP-4-7	2	1	Paraburkholderia sp. RP-4-7 hits
...... Paraburkholderia caledonica	2	1	Paraburkholderia caledonica hits
..... Burkholderia sp. WSM2230	1	1	Burkholderia sp. WSM2230 hits
.... Duganella sp. DN04	2	1	Duganella sp. DN04 hits
... Alphaproteobacteria	27	16	
.... Rhizobiales	25	14	
..... Methylobacterium	11	6	
...... unclassified Methylobacterium	6	3	
.... Methylobacterium sp. 174MFSha1.1	2	1	Methylobacterium sp. 174MFSha1.1 hits
.... Methylobacterium sp. 6HR-1	2	1	Methylobacterium sp. 6HR-1 hits
.... Methylobacterium sp. 17Sr1-43	2	1	Methylobacterium sp. 17Sr1-43 hits
...... Methylobacterium variabile	2	1	Methylobacterium variabile hits

...... Methylobacterium tarhaniae	2	1	Methylobacterium tarhaniae hits
...... Methylobacterium crusticola	1	1	Methylobacterium crusticola hits
..... Phyllobacteriaceae	14	8	
...... Mesorhizobium	12	7	
.... Mesorhizobium ciceri	1	1	Mesorhizobium ciceri hits
.... unclassified Mesorhizobium	11	6	
.... Mesorhizobium sp. B2-3-3	1	1	Mesorhizobium sp. B2-3-3 hits
.... Mesorhizobium sp. B2-3-5	2	1	Mesorhizobium sp. B2-3-5 hits
.... Mesorhizobium sp. B2-4-15	2	1	Mesorhizobium sp. B2-4-15 hits
.... Mesorhizobium sp. B4-1-4	2	1	Mesorhizobium sp. B4-1-4 hits
.... Mesorhizobium sp. B2-4-12	2	1	Mesorhizobium sp. B2-4-12 hits
.... Mesorhizobium sp. B2-4-17	2	1	Mesorhizobium sp. B2-4-17 hits
...... Pseudaminobacter manganicus	2	1	Pseudaminobacter manganicus hits
.... Acetobacter	2	2	
..... Acetobacter nitrogenifigens	1	2	Acetobacter nitrogenifigens hits
...... Acetobacter nitrogenifigens DSM 23921 = LMG 23498	1	1	Acetobacter nitrogenifigens DSM 23921 = LMG 23498 hits
... Gammaproteobacteria	10	8	
.... Rhodanobacter	9	7	
..... Rhodanobacter fulvus	1	2	Rhodanobacter fulvus hits
...... Rhodanobacter fulvus Jip2	1	1	Rhodanobacter fulvus Jip2 hits
..... unclassified Rhodanobacter	2	2	
...... Rhodanobacter sp. OR444	1	1	Rhodanobacter sp. OR444 hits
...... Rhodanobacter sp.	1	1	Rhodanobacter sp. hits
..... Rhodanobacter denitrificans	2	1	Rhodanobacter denitrificans hits
..... Rhodanobacter thiooxydans	2	2	Rhodanobacter thiooxydans hits
...... Rhodanobacter thiooxydans LCS2	1	1	Rhodanobacter thiooxydans LCS2 hits
.... Pseudomonas acidophila	1	1	Pseudomonas acidophila hits
.. Capsulimonas corticalis	2	1	Capsulimonas corticalis hits
